# Responses of reactive oxygen species and methylglyoxal metabolisms to magnesium-deficiency differ greatly among the roots, upper and lower leaves of *Citrus sinensis*

**DOI:** 10.1186/s12870-019-1683-4

**Published:** 2019-02-15

**Authors:** Yan-Tong Cai, Han Zhang, Yi-Ping Qi, Xin Ye, Zeng-Rong Huang, Jiu-Xin Guo, Li-Song Chen, Lin-Tong Yang

**Affiliations:** 1 Institute of Plant Nutritional Physiology and Molecular Biology, College of Resources and Environment, Fujian Agriculture and Forestry University, Fuzhou, 350002 China; 2 Institute of Materia Medica, Fujian Academy of Medical Sciences, Fuzhou, 350001 China; 3 Key Lab of Soil Ecosystem Health and Regulation, Fujian Province University, Fujian Agriculture and Forestry University, Fuzhou, 350002 China; 4 The Higher Educational Key Laboratory of Fujian Province for Soil Ecosystem Health and Regulation, College of Resources and Environment, Fujian Agriculture and Forestry University, Fuzhou, 350002 China

**Keywords:** Antioxidant, *Citrus sinensis*, Magnesium (Mg)-deficiency, Methylglyoxal, Reactive oxygen species, Sulfur metabolism

## Abstract

**Background:**

Magnesium (Mg)-deficiency is one of the most prevalent physiological disorders causing a reduction in *Citrus* yield and quality. ‘Xuegan’ (*Citrus sinensis*) seedlings were irrigated for 16 weeks with nutrient solution containing 2 mM (Mg-sufficiency) or 0 mM (Mg-deficiency) Mg(NO_3_)_2_. Thereafter, we investigated the Mg-deficient effects on gas exchange and chlorophyll a fluorescence in the upper and lower leaves, and Mg, reactive oxygen species (ROS) and methylglyoxal (MG) metabolisms in the roots, lower and upper leaves. The specific objectives were to corroborate the hypothesis that the responses of ROS and MG metabolisms to Mg-deficiency were greater in the lower leaves than those in the upper leaves, and different between the leaves and roots.

**Results:**

Mg level was higher in the Mg-deficient upper leaves than that in the Mg-deficient lower leaves. This might be responsible for the Mg-deficiency-induced larger alterations of all the measured parameters in the lower leaves than those in the upper leaves, but they showed similar change patterns between the Mg-deficient lower and upper leaves. Accordingly, Mg-deficiency increased greatly their differences between the lower and upper leaves. Most of parameters involved in ROS and MG metabolisms had similar variation trends and degrees between the Mg-deficient lower leaves and roots, but several parameters (namely glutathione S-transferase, sulfite reductase, ascorbate and dehydroascorbate) displayed the opposite variation trends. Obviously, differences existed in the Mg-deficiency-induced alterations of ROS and MG metabolisms between the lower leaves and roots. Although the activities of most antioxidant and sulfur metabolism-related enzymes and glyoxalase I and the level of reduced glutathione in the Mg-deficient leaves and roots and the level of ascorbate in the leaves were kept in higher levels, the levels of malonaldehyde and MG and/or electrolyte leakage were increased in the Mg-deficient lower and upper leaves and roots, especially in the Mg-deficient lower leaves and roots.

**Conclusions:**

The ROS and MG detoxification systems as a whole did not provide sufficient detoxification capacity to prevent the Mg-deficiency-induced production and accumulation of ROS and MG, thus leading to lipid peroxidation and the loss of plasma membrane integrity, especially in the lower leaves and roots.

**Electronic supplementary material:**

The online version of this article (10.1186/s12870-019-1683-4) contains supplementary material, which is available to authorized users.

## Background

Magnesium (Mg), an essential macronutrient for the normal growth and development of higher plants, plays key roles in various biochemical and physiological processes, including chlorophyll (Chl) biosynthesis, gas exchange, and formation and detoxification of reactive oxygen species (ROS) [1–7]. Despite all this, Mg has been neglected by researchers in plant nutrition compared with the other macronutrients [8]. Mg-deficiency is becoming an increasingly serious and urgent problem adversely affecting productivity and quality of many agricultural crops [9, 10]. In China, Mg-deficiency is one of the most common physiological disorders causing a reduction in yield and quality of *Citrus* [11, 12].

Mg-deficiency-induced decline in leaf CO_2_ assimilation is a very common phenomenon occurred in many plants including *Citrus* [12–17]. A consequence of the decline in photosynthesis in response to Mg-deficiency is that less of the absorbed photon-energy captured by light-harvesting pigments is utilized in the photosynthetic electron transport, so more excess light energy, which can potentially induce the formation of ROS [18], exists in the Mg-deficient leaves [2, 14]. Plants have evolved diverse enzymatic [namely guaiacol peroxidase (GuPX), ascorbate (ASC) peroxidase (APX), superoxide dismutase (SOD), monodehydroascorbate (MDHA) reductase (MDHAR), dehydroascorbate (DHA) reductase (DHAR), glutathione reductase (GR), catalase (CAT) and sulfur (S) metabolism-related enzymes] and non-enzymatic [namely reduced glutathione (GSH) and ASC] detoxification systems to protect cells against oxidative stress due to increased production and accumulation of ROS [18–20]. Antioxidant enzyme system has been considered as the first line of defense against the oxidative stress [21]. Mg-deficiency-induced increases of both antioxidant enzyme activities and antioxidant metabolite levels in leaves have been observed in many plants, including: bean [22, 23], maize [24], wheat [25], mulberry [26], *Citrus* [2, 14], rice [27, 28], and pepper [29]. There are reports showing that the Mg-deficiency-induced upregulation of both antioxidant metabolites and enzymes may provide sufficient protection to them against oxidative stress, as indicated by the unaltered or decreased malondialdehyde (MDA) level in the Mg-deficient leaves of *Citrus reticulata* [14], rice [28] and mulberry [26]. However, MDA level was elevated in the Mg-deficient leaves of maize [24], rice [27], *Citrus grandis* and *Citrus sinensis* [2] accompanied by enhanced antioxidant capacity.

Thiol-based antioxidant system is the second line of defense against the oxidative damage [21]. Evidence shows that S metabolism, a core pathway for the biosynthesis of S-containing compounds-namely cysteine (Cys), GSH and H_2_S, plays crucial roles in plant adaptive responses to abiotic stresses [19, 20, 30]. So far, a few studies have investigated the responses of S metabolism to nutrient deficiencies. Most of these studies have focused on iron (Fe) and nitrogen (N) deficiencies [31–33]. Very little is known about the Mg-deficiency-induced alterations of S metabolism in plant leaves. Elevated concentration of GSH (SH-compounds) and/or ratio of GSH/oxidized glutathione (GSSG) have been observed in Mg-deficient leaves of bean [23] and rice [28]. Yang et al. [2] reported that the Mg-deficient *C. grandis* and *C. sinensis* leaves had higher GSSG level, lower GSH level and GSH/(GSH + GSSG) ratio, but unaltered (GSH + GSSG) level. The Mg-deficient pepper leaves displayed a relatively lower ratio of GSH/GSSG [29]. Thus, it is reasonable to hypothesize that the activities of S metabolism-related enzymes [i.e., ATP sulphurylase (ATPS), adenosine 5′-phosphosulfate reductase (APR), Cys synthase (CS), glutathione S-transferase (GST), γ-glutamylcysteine synthase (γGCS), sulfite reductase (SiR) and γ-glutamyltransferase (γGT)] might be altered in the Mg-deficient leaves.

Production and accumulation of methylglyoxal (MG, a cytotoxic compound) in plant cells often increases in response to abiotic stresses [20, 34, 35]. Over-accumulation of MG in plant cells can lead to detrimental effects by promoting ROS generation and inhibiting antioxidant enzyme system [34, 36]. To mitigate cellular injury caused by increased accumulation of MG, plants have evolved a MG-detoxifying glyoxalase (Gly) system, mainly including Gly I and Gly II. Gly I converts hemithioacetal (HTA), formed spontaneously from MG and GSH, to S-D-lactoylglutathione (SLG). SLG is then hydrolyzed to D-lactate by Gly II and one molecule of GSH is recycled back in the Gly system. Thus, the availability of GSH plays a key role in the detoxification of MG [33, 34]. Studies demonstrated that several metabolic pathways (i.e., photosynthesis, respiration, glycolysis and membrane lipid peroxidation) involved in the production of MG were affected in the Mg-deficient leaves [2–4, 7, 14, 24]. In a study, Peng et al. [37] observed that Mg-deficiency decreased the abundance of chloroplastic Gly I in *C. sinensis* leaves. Therefore, the production and accumulation of MG, as well as the activities of glyoxalases should be altered in Mg-deficient leaves.

In Mg-starved plants, Mg remobilization from the old leaves to the young tissues was increased [38]. Previous studies showed that Mg-deficiency affected pigments, gas exchange, organic acid (OA), protein, amino acid and carbohydrate metabolisms more in the older leaves than those in the younger leaves [3, 39, 40]. This drives us to hypothesize that the Mg-deficiency-induced alterations of ROS and MG metabolism might become more pronounced with increasing leaf age. Few studies investigated the Mg-deficient effects on the concentrations of antioxidant metabolites, the activities of antioxidant enzymes, and lipid peroxidation in the leaves of different positions (ages) [29, 40–42], but the results were somewhat inconsistent. In a study with common bean plants, Huang and Chu [40] found that the Mg-deficiency-induced increases of electrolyte leakage, MDA concentration and APX activity were greater in the older primary leaves than those in the younger first trifoliate leaves, and that the activities of SOD, GuPX and CAT were not unaltered in the Mg-deficient primary leaf, but increased in the Mg-deficient first trifoliate leaf except for unchanged CAT activity, demonstrating that ROS metabolism was less affected in the first trifoliate leaf than in the primary leaf. However, Anza et al. [29] observed that the Mg-deficiency-induced antioxidant responses were less in the youngest and the oldest leaves. To our knowledge, very little is known about the Mg-deficient effects on the activities of enzymes related to S metabolism and MG detoxification in the leaves of different positions (ages).

So far, most of studies have focused on Mg-deficient effects on leaves because Mg-deficiency-induced leaf chlorosis is one of the earliest and the most easily identified symptoms [2, 43], less was known about the Mg-deficient effects on the roots [3, 44]. There are several reports showing that the Mg-deficient effects on anatomy, gas exchange, carbohydrate, OA, amino acid and phenolic metabolisms differ between the roots and leaves [2–4, 6, 7, 45–47]. Transcriptome, proteome and miRNA analyses reveal that the Mg-deficient effects on the expression of genes, proteins and miRNAs involved in carbohydrate and energy metabolism, ROS and MG detoxification, and amino acid and protein metabolisms differ between the roots and leaves [4, 37, 48–51]. Thus, the responses of ROS and MG metabolisms to Mg-deficiency should differ between the leaves and roots.

Here, we investigated the Mg-deficiency-induced alterations of gas exchange and Chl a fluorescence in the lower and upper leaves, and Mg, ROS production, electrolyte leakage, MDA, MG, antioxidant metabolites, and enzymes involved in ROS and MG detoxification in the roots, lower and upper leaves of *C. sinensis* seedlings. Our specific objectives were to corroborate the hypothesis that the Mg-deficiency-induced alterations of ROS and MG metabolisms were greater in the lower leaves than those in the upper leaves, and different between the leaves and roots.

## Methods

### Seedling culture and mg treatments

Seedling culture and Mg treatments were performed according to Peng et al. [37] with some modifications. Fifteen week-old seedlings of *C. sinensis* cv. Xuegan in 6 L pots (two seedlings per pot) containing clean river sand washed thoroughly with tap water were raised in a greenhouse under natural photoperiod at Fujian Agriculture and Forestry University, Fuzhou, China (26° 5’ N, 119° 14′ E). Each pot were fertilized every 2 day with nutrient solution containing 1 mM KNO_3_, 2 mM K_2_SO_4_, 5 mM Ca(NO_3_)_2_, 1 mM KH_2_PO_4_, 10 μM H_3_BO_3_, 2 μM MnCl_2_, 2 μM ZnSO_4_, 0.5 μM CuSO_4_, 0.065 μM (NH_4_)_6_Mo_7_O_24_, 20 μM Fe-EDTA and 0 mM or 2 mM Mg(NO_3_)_2_ until some of nutrient solution flowed out of the hole in the bottom of the pot (~ 500 mL). To maintain a constant N concentration, equivalent moles of NH_4_NH_3_ instead of Mg(NO_3_)_2_ were added in the nutrient solution. Sixteen weeks after Mg treatments, ~ 5-mm-long white root tips, and ~ 11-week-old lower (quarter height) and ~ 5-week-old upper (three quarter height) leaves were used for all measurements with the exception of root Mg concentration [3]. Leaves (midribs and petioles removed), leaf disks (0.6 cm in diameter) and ~ 5-mm-long white root tips were collected from the plants that had been used for the measurements of leaf gas exchange and Chl a fluorescence at noon in the sunny day. After being frozen immediately in liquid N_2_, all samples were stored at − 80 °C until extraction of enzymes, total soluble proteins, antioxidant metabolites, MDA and MG. These unsampled seedlings were used to measure Mg, electrolyte leakage, and superoxide anion and H_2_O_2_ generation.

### Root and leaf mg

The small (< 2 mm in diameter) first- and second-order fibrous roots [20], upper and lower leaves (midribs and petioles removed) [3] were used for the measurements of Mg. Root and leaf Mg concentration was measured using PinAAcle 900F Atomic Absorption Spectrometer (PerkinElmer Singapore Pte Ltd., Singapore) [3]. There were 10 replicates per treatment.

### Leaf gas exchange and Chl a fluorescence

Gas exchange was measured with a portable photosynthesis system (CIRAS-2, PP-Systems, Herts, UK) at a leaf temperature of 27.79 ± 0.3 °C, a relative humidity of 46.1 ± 1.2%, a controlled CO_2_ concentration of ~ 380 μmol mol^− 1^ and a controlled light intensity of ~ 1000 μmol m^− 2^ s^− 1^ between 9 and 11 a.m. on a sunny day. Water use efficiency (WUE) was the ratio of leaf CO_2_ assimilation to transpiration. There were 6 replicates per treatment.

Chl a fluorescence (OJIP) transients were measured after the seedlings were dark-adapted for 3 h at room temperature with a Handy PEA (Hansatech Instruments Limited, Norfolk, UK) as described by Jiang et al. [52]. OJIP transient was analyzed according to JIP test. Specific energy fluxes per reaction center (RC) for energy dissipation (DI_o_/RC), maximum photosystem II (PSII) efficiency of dark-adapted leaves (F_v_/F_m_), quantum yield for energy dissipation (DI_o_/ABS), the fraction of oxygen-evolving complexes (OEC) in comparison with control, and total performance index (PI_tot,abs_) were calculated according to Jiang et al. [52] and Liao et al. [53]. There were 8 replicates per treatment.

### Electrolyte leakage, generation rates of H_2_O_2_ and superoxide anion, concentrations of total soluble proteins, MDA, MG and antioxidants in leaves and roots

Electrolyte leakage was measured as described by Long et al. [54]. H_2_O_2_ and superoxide anion generationrates were determined as described previously [55]. Total soluble proteins were measured according to Bradford [56]. MDA was determined spectrophotometrically after being extracted with 80% (v/v) ethanol [57]. MG was assayed with an *N*-acetyl-l-Cys assay according to Wild et al. [58] after being extracted by 5% (v/v) HClO_4_. GSH and GSSG, and ASC and DHA were assayed according to Chen et al. [55] after being extracted with 5% (w/v) trichloroacetic acid (TCA) and 6% (v/v) of HClO_4_, respectively. There were 8 replicates per treatment except for 4 replicates for root H_2_O_2_ production rate.

### Enzyme activities in leaves and roots

Glutathione peroxidase (GlPX), SOD, APX, MDHAR, DHAR, GR, CAT, GuPX and GST were extracted with 50 mM KH_2_PO_4_-KOH (pH 7.5) containing 0.1 mM EDTA, 0.3% (w/v) Triton X-100 and 4% (w/v) insoluble polyvinylpolypyrrolidone (PVPP) [18]. SOD and GuPX were assayed according to Giannopolitis and Ries [59] and Chen et al. [60], respectively. CAT, GR, MDHAR, DHAR and APX were determined according to Chen and Cheng [18]. GlPX and GST were assayed according to Guo et al. (20). There were 8 replicates per treatment.

APR, CS, ATPS, SiR, γGT, γGCS, Gly I and Gly II were extracted according to Lappartient and Touraine [61]. Briefly, six 6-mm-diameter frozen leaf discs or ~ 100 mg frozen root apices were ground with a precooled mortar and pestle in 1 mL ice-cold extraction buffer containing100 mM Tris-HCl (pH 8.0), 10 mM EDTA, 2 mM dithiothreitol (DTT) and 4% (w/v) insoluble PVPP. ATPS and CS activities were measured according to Guo et al. [20] and Warrilow and Hawkesford [62], respectively. APR activity was determined according to Trüper and Rogers [63] with some modification. Briefly, 1 mL reaction mixture contained 50 mM Tris-HCl (pH 8.0), 0.5 mM K_3_Fe(CN)_6_, 8 mM EDTA, 0.4 mM AMP, 4 mM Na_2_SO_3_ and 100 μL extract. SiR were assayed in 1 mL reaction mixture containing 10 mM Tris-HCl (pH 7.5), 0.1 mM EDTA, 0.5 mM Na_2_SO_3_, 0.2 mM NADPH and 100 μL enzyme extract [64]. γGT was assayed as described previously [65]. Briefly, 1 mL reaction mixture contained 100 mM Tris-HCl (pH 8.0), 20 mM glycylglycine (Gly-Gly), 2.5 mM l-γ-glutamyl-p-nitroanilide and 100 μL enzyme extract. After 10 min incubation at 25 °C, the reaction was stopped by the addition of 1 mL of 25% (w/v) TCA. The resultant p-nitroaniline (ε = 1.74 mM^− 1^ cm^− 1^) was measured at 405 nm. γGCS was assayed in 1 mL reaction buffer containing 100 mM Tris-HCl (pH 8.0), 20 mM MgCl_2_, 150 mM KCl, 2 mM EDTA, 2 mM phosphoenolpyruvate (PEP), 5 mM ATP, 10 mM glutamate, 10 mM α-aminobutyrate, 0.2 mM NADH, 7 U pyruvate kinase (PK), 10 U lactate dehydrogenase (LDH) and 100 μL enzyme extract [65]. Gly I and Gly II activities were assayed as described previously [66]. There were 8 replicates per treatment.

Glutamine synthetase (GS) was extracted and assayed as previously [20]. There were 6 replicates for leaf GS and 4 replicates for root GS per treatment.

### Data analysis

There were 20 pots (40 seedlings) per treatment in a completely randomized design. Results were expressed as mean ± SE (*n* = 4–10). Significant differences between Mg-deficient roots and controls were made by unpaired *t*-test. Four means [two (Mg levels) × two (leaf positions)] were analyzed by two ANOVA followed by Duncan’s new multiple range test.

Principal component analysis (PCA) and Pearson correlation analysis for all the measured parameters except for leaf gas exchange and Chl a fluorescence parameters were performed using a SPSS® statistical software (version 17.0, IBM, NY, USA), as described previously [20, 37].

## Results

### Typical Mg-deficient symptoms occurred only in Mg-deficient lower leaves, and Mg-deficiency affected Mg concentration more in leaves than in roots

**Fig. 1:**
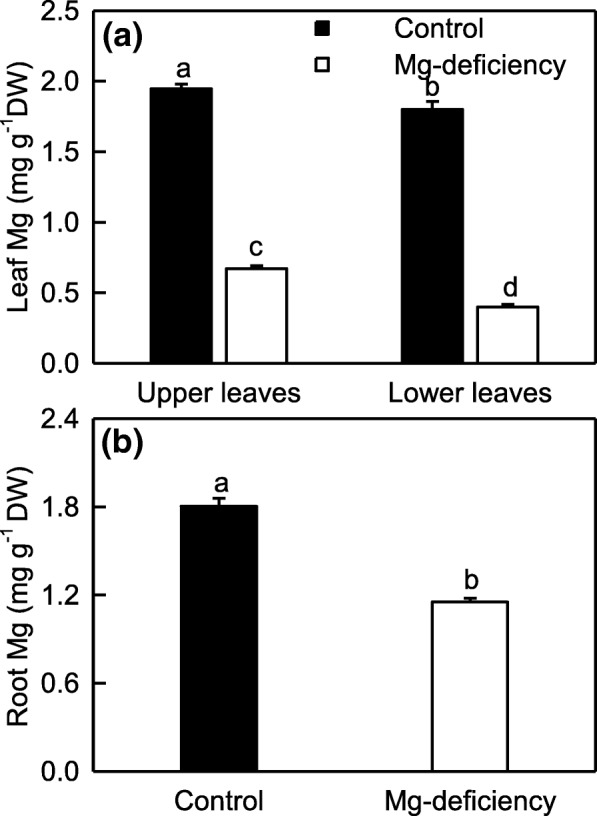
Mg-deficient effects on the concentration of Mg in *C. sinensis* leaves (**a**) and roots (**b**). Bars represent means ± SE (*n* = 10). Significant differences between Mg-deficient roots and controls were made by unpaired *t*-test. Four means [two (Mg levels) × two (leaf positions)] were analyzed by two ANOVA followed by Duncan’s new multiple range test. Different letters above the bars indicate a significant difference at *P* < 0.05

In addition to inhibiting seedling growth, 0 mM Mg-treatment led to a typical Mg-deficient system (leaf chlorosis) in the basal older leaves. The symptom first occurred in the old leaves, and then extended gradually to the young leaves with the prolongation of Mg-deficiency duration (Additional file 1: Fig. S1). Similar to *Citrus* boron (B)-deficiency [3, 67], enlargement and corkiness of midrib and main lateral veins were observed in 0 mM Mg-treated old (lower) leaves, but not in the upper leaves. Seedlings supplied with 2 mM Mg did not display any Mg-deficient symptoms (Additional file 1: Figure S1). Also, foliar Mg level (Fig. 1a) fell in the normal range [43]. Therefore, plants exposed to 2 mM and 0 mM Mg are considered as Mg-sufficient (control) and Mg-deficient, respectively.

Mg-deficiency-induced decrease in Mg concentration was in the order of lower leaves > upper leaves > roots. The lower leaves had less Mg concentration than the upper leaves under the same concentration of Mg supply (Fig. 1).

### Gas exchange and Chl a fluorescence parameters were greatly altered in the Mg-deficient lower leaves, but not in the Mg-deficient upper leaves with the exception of a few

**Fig. 2:**
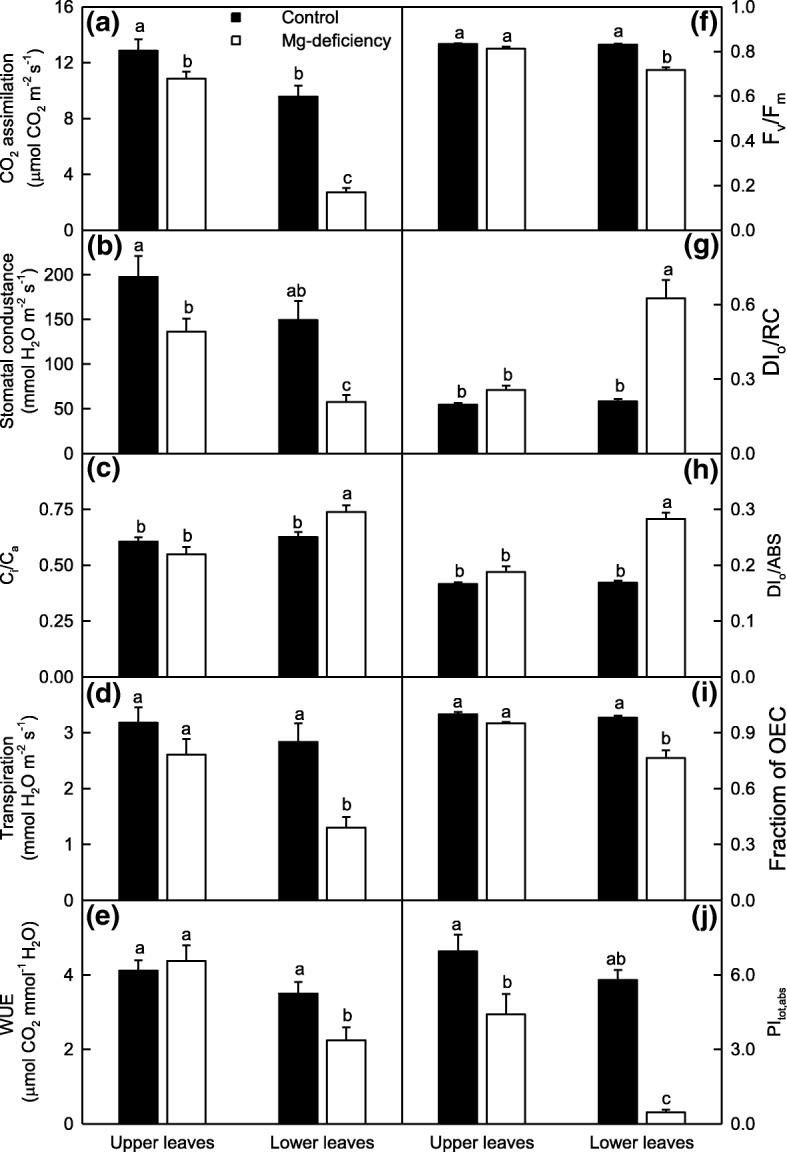
Mg-deficient effects on CO_2_ assimilation (**a**), stomatal conductance (g_s_, **b**), the ratio of intercellular to ambient CO_2_ concentration (C_i_/C_a_; **c**), transpiration (**d**), water use efficiency (WUE, **e**), F_v_/F_m_ (**f**), DI_o_/RC (**g**), DI_o_/ABS (**h**), the fraction of OEC in comparison with control (**i**) and PI_tot,abs_ (**j**) in *C. sinensis* leaves. Bars represent means ± SE (*n* = 6 for gas exchange or 8 for fluorescence parameters). Significant differences between Mg-deficient roots and controls were made by unpaired *t*-test. Four means [two (Mg levels) × two (leaf positions)] were analyzed by two ANOVA followed by Duncan’s new multiple range test. Different letters above the bars indicate a significant difference at *P* < 0.05

As shown in Fig. 2, the Mg-deficient lower leaves had decreased CO_2_ assimilation, stomatal conductance (g_s_), transpiration, WUE, F_v_/F_m_, the fraction of OEC in comparison with control and PI_tot,abs_, but increased ratio of intercellular to ambient CO_2_ concentration (C_i_/C_a_), DI_o_/RC and DI_o_/ABS in the lower leaves. However, only CO_2_ assimilation, g_s_ and PI_tot,abs_ were significantly decreased in the Mg-deficient upper leaves. Thus, it is reasonable to assume that the Mg-deficiency-induced alterations of leaf gas exchange and fluorescence parameters increased with increasing ages. Under Mg-deficiency, the upper leaves had higher CO_2_ assimilation, g_s_, transpiration, WUE, F_v_/F_m_, the fraction of OEC in comparison with control and PI_tot,abs_ than the lower leaves, but lower C_i_/C_a_, DI_o_/RC and DI_o_/ABS. Under Mg-sufficiency, all the ten parameters were similar between the upper and lower leaves except that CO_2_ assimilation was slightly higher in the upper leaves than that in the lower leaves. Obviously, Mg-deficiency increased greatly the differences in gas exchange and fluorescence parameters between the upper and lower leaves.

### Electrolyte leakage, superoxide anion and H_2_O_2_ generation rates, and MDA and MG levels were greatly increased in the Mg-deficient lower leaves and roots, but not in the Mg-deficient upper leaves

**Fig. 3:**
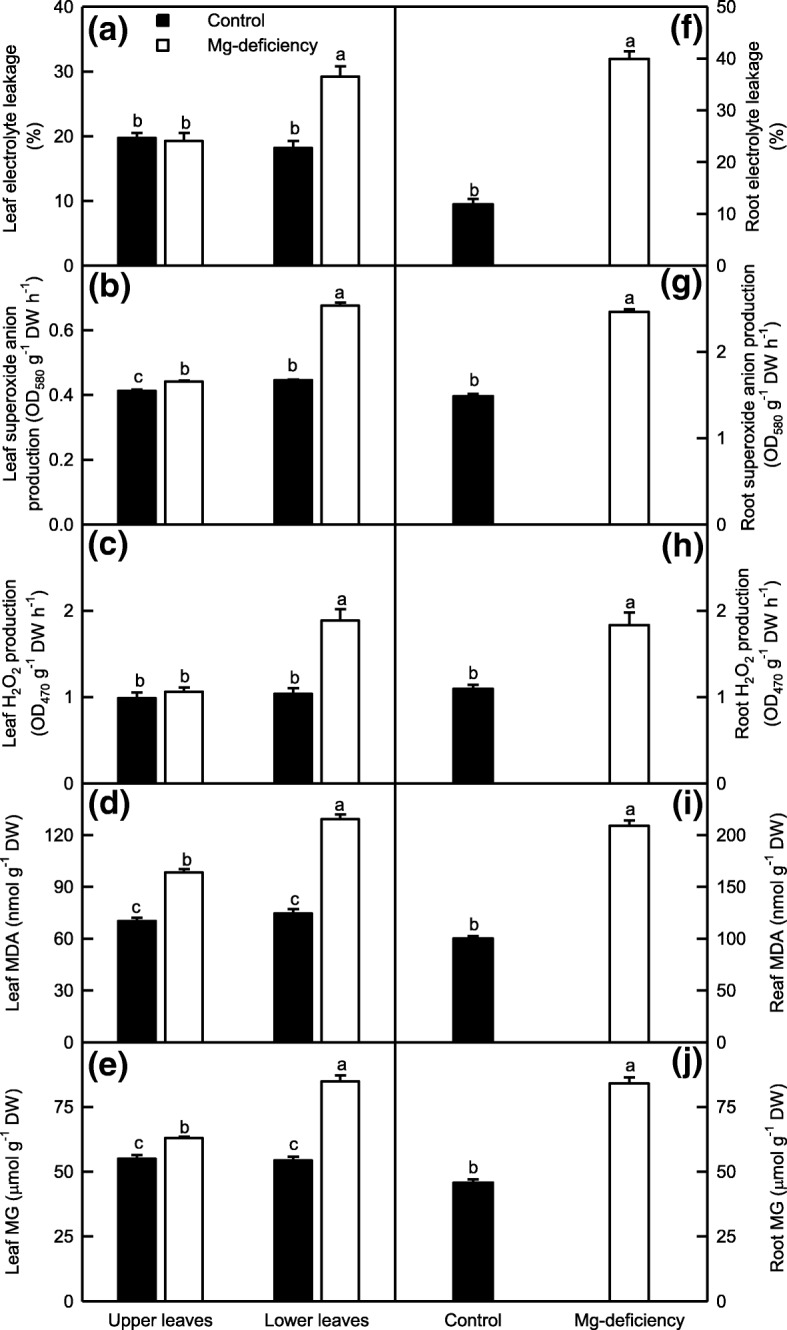
Mg-deficient effects on electrolyte leakage (**a, f**), superoxide anion (**b, g**) and H_2_O_2_ (**c, h**) production rates, MDA (**d, i**) and MG (**e, j**) concentrations in *C. sinensis* leaves (**a-e**) and roots (**f-j**). Bars represent means ± SE (*n* = 8 except for 4 for root H_2_O_2_ production rate). Significant differences between Mg-deficient roots and controls were made by unpaired *t*-test. Four means [two (Mg levels) × two (leaf positions)] were analyzed by two ANOVA followed by Duncan’s new multiple range test. Different letters above the bars indicate a significant difference at *P* < 0.05

Since CO_2_ assimilation was greatly inhibited in the Mg-deficient lower leaves, but only slightly inhibited in the Mg-deficient upper leaves (Fig. 2a), the fraction of the absorbed light allocated to photosynthetic electron transport might be greatly decreased only in the Mg-deficient lower leaves. As a result, more excess light energy might exist in the Mg-deficient lower leaves, as indicated by the increased DI_o_/ABS and DI_o_/RC, but not in the Mg-deficient upper leaves, as indicated by the unaltered DI_o_/ABS and DI_o_/RC (Fig. 2f-h). The excess absorbed light energy can potentially promote the production of ROS and MG, thus impairing redox homeostasis and causing lipid peroxidation [18, 20]. Lipid peroxidation may lead to the loss of membrane integrity and the increase of electrolyte leakage [19, 20, 34, 35]. For this purpose, we investigated the Mg-deficient effects on electrolyte leakage, superoxide anion and H_2_O_2_ generation rates, and MDA and MG levels in the upper and lower leaves. All the five parameters were greatly increased in the Mg-deficient lower leaves, but not in the Mg-deficient upper leaves except for a slight increase in H_2_O_2_ production rate. Thus, all the five parameters did not significantly differ between the upper and lower leaves except for a slightly higher H_2_O_2_ production rate in the lower leaves under Mg-sufficiency, but they were higher in the lower leaves than those in the upper leaves under Mg-deficiency (Fig. 3a-e). Like to the lower leaves, all the five parameters were greatly elevated in the Mg-deficient roots (Fig. 3f-j).

### Relationships between CO_2_ assimilation, Chl a fluorescence parameters, electrolyte leakage, ROS production rates, MDA and MG levels in leaves

We calculated the linear correlation coefficients between CO_2_ assimilation, F_v_/F_m_, DI_o_/RC, DI_o_/ABS, the fraction of OEC in comparison with control, PI_tot,abs_, electrolyte leakage, ROS production rates, MDA and MG levels in leaves in order to understand the relationships between them (Table 1). Most of these physiological parameters were positively or negatively related with each other. Indeed, only ten correlation coefficients (a total of 55) were not significant.

**Table 1:** Pearson correlation coefficient matrix for 11 physiological parameters in *C. sinensis* leaves. Data are from Figs. 2 and 3; *, ** and *** indicate a significant difference at *P* < 0.05, *P* < 0.01 and *P* < 0.001, respectively. A: CO_2_ assimilation; F_OEC_: The fraction of OEC in comparison with control; EL: Electrolyte leakage; SAP: Superoxide anion production rate; HP: H_2_O_2_ production rate

	A	F_v_/F_m_	DI_o_/RC	DI_o_/ABS	F_OEC_	PI_tot,abs_	EL	SAP	HP	MDA	MG
A	1										
F_v_/F_m_	0.9492	1									
DI_o_/RC	−0.9569*	−0.9987**	1								
DI_o_/ABS	−0.9489	−1.0000***	0.9986**	1							
F_OEC_	0.9629*	0.9986**	−0.9976**	−0.9985**	1						
PI_tot,abs_	0.9484	0.9754*	−0.9673*	−0.9755*	0.9823*	1					
EL	−0.9080	−0.9798*	0.9845*	0.9797*	−0.9706*	−0.9116	1				
SAP	−0.9806*	−0.9876*	0.9934**	0.9874*	−0.9910**	−0.9573*	0.9723*	1			
HP	−0.9656*	−0.9924**	0.9974**	0.9922**	−0.9922**	−0.9529*	0.9854*	0.9978**	1		
MDA	−0.8838	−0.9550*	0.9395	0.9553*	−0.9569*	−0.9863*	0.8869	0.9114	0.9141	1	
MG	−0.9202	−0.9941**	0.9873*	0.9942**	−0.9910**	−0.9809*	0.9643*	0.9655*	0.9731*	0.9776*	1

### Mg-deficiency-induced changes in the activities of enzymes involved in ROS and MG detoxification were more pronounced in the lower leaves and roots than in the upper leaves

**Fig. 4:**
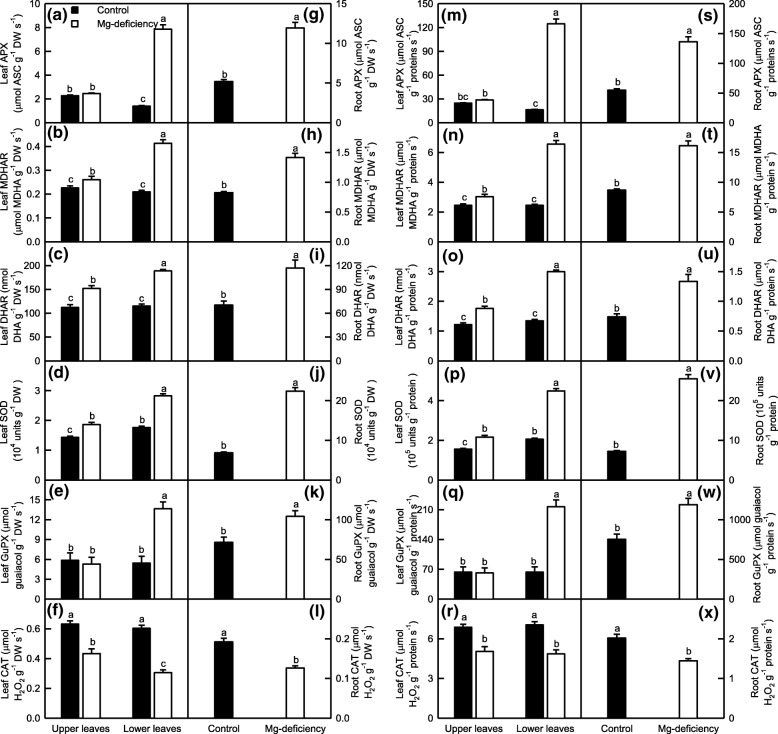
Mg-deficient effects on APX (**a, g, m** and **s**), MDHAR (**b, h, n** and **t**), DHAR (**c, i, o** and **u**), SOD (**d, j, p** and **v**), GuPX (**e, k, q** and **w**) and CAT (**f, l, r** and **x**) activities expressed on a DW (**a-l**) or protein (**m-x**) basis in *C. sinensis* leaves (**a-f** and **m-r**) and roots (**g-l** and **s-x**). Bars represent means ± SE (*n* = 8). Significant differences between Mg-deficient roots and controls were made by unpaired *t*-test. Four means [two (Mg levels) × two (leaf positions)] were analyzed by two ANOVA followed by Duncan’s new multiple range test. Different letters above the bars indicate a significant difference at *P* < 0.05

To deal with oxidative injury, plants have evolved efficient enzymatic and non-enzymatic scavenging systems. Antioxidant enzymes are the first line of defense against the oxidative injury [21]. As shown in Fig. 4, Mg-deficiency increased the activities of APX, MDHAR, DHAR, SOD and GuPX in the roots and lower leaves whether the data were expressed on a DW or protein basis, but had less influence on them in the upper leaves. By contrast, CAT activity was reduced in the Mg-deficient leaves and roots. Under Mg-deficiency, the activities of APX, MDHAR, DHAR, SOD and GuPX were higher in the lower leaves than those in the upper leaves, whereas the activity of CAT was higher in the upper leaves than that in the lower leaves or similar between the two depending on how the data were expressed. Under Mg-sufficiency, the activities of all the six antioxidant enzymes were similar between the upper and lower leaves except that SOD activity (APX activity on a DW basis) was slightly higher (less) in the lower leaves than that in the upper leaves.

**Fig. 5:**
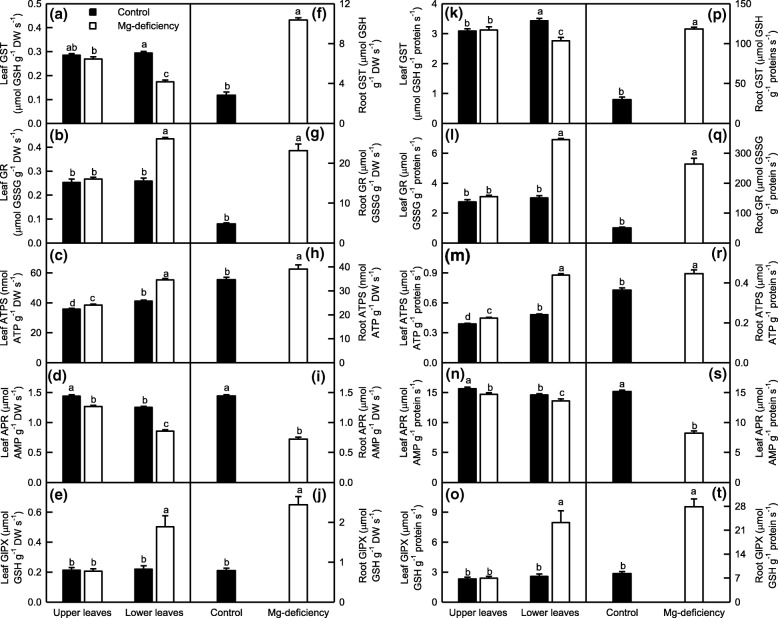
Mg-deficient effects on GST (**a, f, k** and **p**), GR (**b, g, l** and **q**), ATPS (**c, h, m** and **r**), APR (**d, i, n** and **s**) and GlPX (**e, j, o** and **t**) activities expressed on a DW (**a-j**) or protein (**k-t**) basis in *C. sinensis* leaves (**a**-**e** and **k**-**o**) and roots (**f-j** and **p-t**). Bars represent means ± SE (*n* = 8). Significant differences between Mg-deficient roots and controls were made by unpaired *t*-test. Four means [two (Mg levels) × two (leaf positions)] were analyzed by two ANOVA followed by Duncan’s new multiple range test. Different letters above the bars indicate a significant difference at *P* < 0.05

**Fig. 6:**
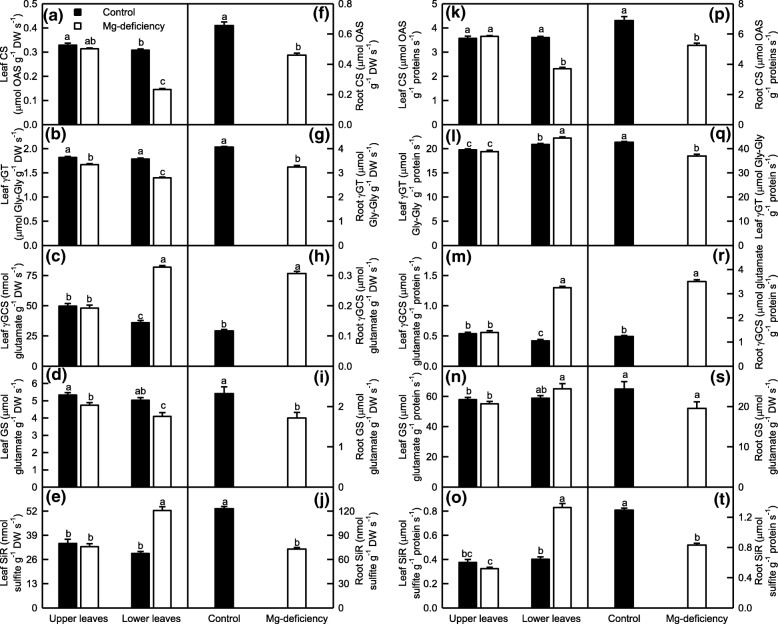
Mg-deficient effects on CS **(a, f, k** and **p**), γGT (b**, g, l** and **q**), γGCS (**c, h, m** and **r**), GS (**d, i, n** and **s**) and SiR (**e**, **j**, **o** and **t**) activities expressed on a DW (**a-j**) or protein (**k-t**) basis in *C. sinensis* leaves (**a-e** and **k-o**) and roots (**f-j** and **p-t**). Bars represent means ± SE (*n* = 8 except for 6 for leaf GS and 4 for root GS). Significant differences between Mg-deficient roots and controls were made by unpaired *t*-test. Four means [two (Mg levels) × two (leaf positions)] were analyzed by two ANOVA followed by Duncan’s new multiple range test. Different letters above the bars indicate a significant difference at *P* < 0.05. OAS: O-acetyl-L-serine

Thiol-based antioxidant system is the second line of defense against the oxidative injury [21]. In the lower leaves, Mg-deficiency decreased the activities of GST, APR and CS on a DW or protein basis and of γGT and GS on a DW basis, increased the activities of GR, ATPS, GlPX, γGCS and SiR on a DW or protein basis and of γGT on a protein basis, and did not affect the activity of GS on a protein basis. In the upper leaves, Mg-deficiency did not affect the activities of all the ten enzymes related to S metabolism except that Mg-deficiency slightly increased the activity of ATPS, and slightly decreased the activities of APR on a DW or protein basis and of γGT and GS on a DW basis. Under Mg-deficiency, the activities of GST, APR, CS on a DW or protein basis and of γGT and GS on a DW basis were higher in the upper leaves than those in the lower leaves, whereas the reverse was the case for the activities of GR, ATPS, GlPX, γGCS and SiR on a DW or protein basis and of γGT and GS on a protein basis. Under Mg-sufficiency, the activities of all the ten S metabolism-related enzymes did not significantly differ between the lower and upper leaves with the exceptions that the activities of ATPS on a DW or protein basis and of GST and γGT on a protein basis were slightly higher in the lower leaves than those in the upper leaves, and that the activities of APR and γGCS on a DW or protein basis and of CS on a DW basis were slightly higher in the upper leaves than those in the lower leaves. In the roots, Mg-deficiency increased the activities of GST, GR, ATPS, GlPX and γGCS, and decreased the activities of the other five enzymes, regardless of how the data were expressed (Figs. 5-6).

**Fig. 7:**
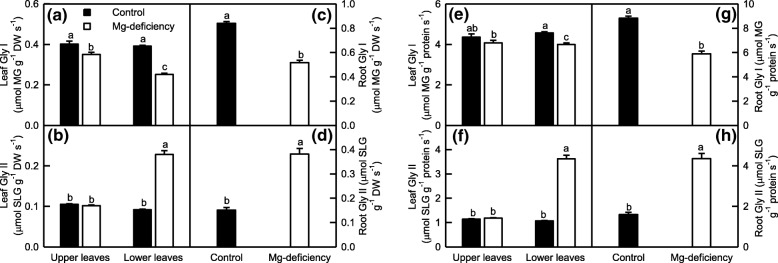
Mg-deficient effects on Gly I (**a, c, e** and **g**) and Gly II (**b, d, f** and **h**) activities expressed on a DW (**a-d**) or protein (**e-h**) basis in *C. sinensis* leaves (**a-b** and **e-f**) and roots (**c-d** and **g-h**). Bars represent means ± SE (*n* = 8). Significant differences between Mg-deficient roots and controls were made by unpaired *t*-test. Four means [two (Mg levels) × two (leaf positions)] were analyzed by two ANOVA followed by Duncan’s new multiple range test. Different letters above the bars indicate a significant difference at *P* < 0.05

Both Gly I and Gly II play a key role in the detoxification of MG [35]. As shown in Fig. 7, Mg-deficiency led to a decreased activity of Gly I and an increased activity of Gly II in the lower leaves and roots whether the data were expressed on a DW or protein basis, but did not affect their activities in the upper leaves except that Mg-deficiency slightly decreased the activity of Gly I on a DW basis. Under Mg-deficiency, the activity of Gly I was higher in the upper leaves than that in the lower leaves, whereas the reverse was the case for the activity of Gly II. Under Mg-sufficiency, the activities of Gly I and Gly II were similar between the upper and lower leaves.

To conclude, Mg-deficiency altered greatly the activities of enzymes involved in ROS and MG detoxification in the lower leaves and roots, but not in the upper leaves with a few of exceptions. The activities of these enzymes differed greatly between the Mg-deficient lower and upper leaves, but not between the Mg-sufficient lower and upper leaves.

### Mg-deficiency-induced alterations of antioxidants were greater in the Mg-deficient lower leaves and roots than in the Mg-deficient upper leaves

**Fig. 8:**
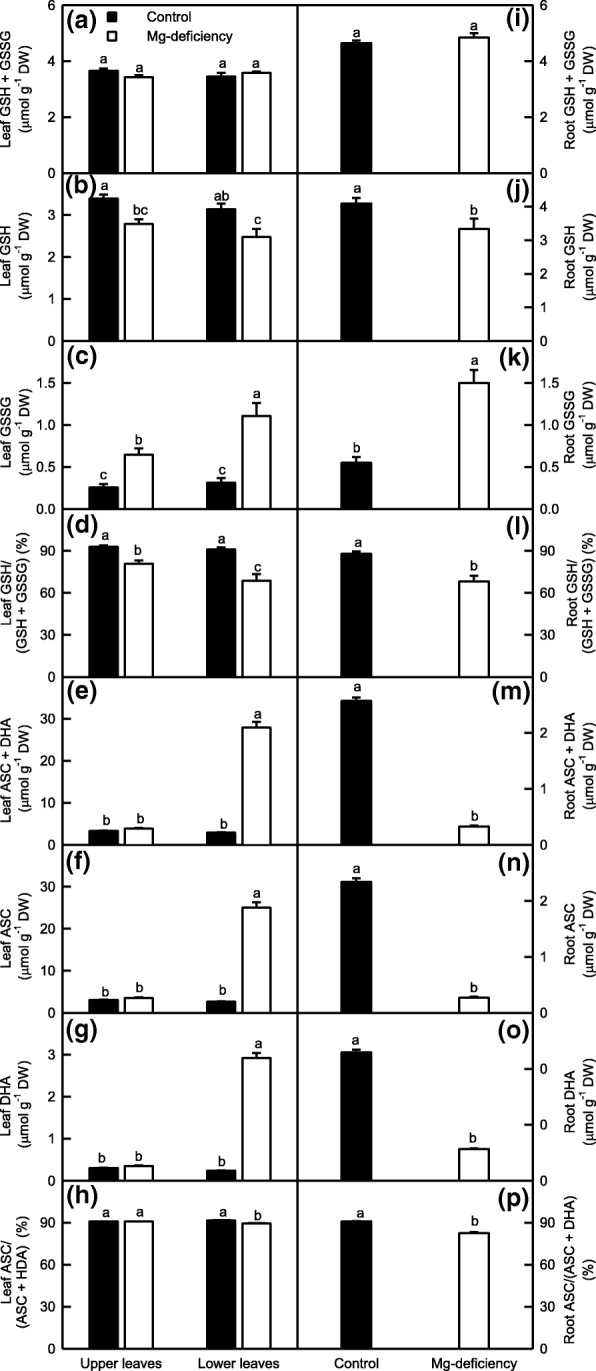
Mg-deficient effects on GSH + GSSG (**a, i**), GSH (**b, j**) and GSSG (**c, k**) concentrations, and GSH/(GSH + GSSG) ratio (**d, l**), and ASC + DHA (**e, m**), ASC (**f, n**) and DHA (**g, o**) concentrations, and ASC/(ASC + DHA) ratio (**h, p**) in *C. sinensis* leaves (**a-h**) and roots (**i-p**). Bars represent means ± SE (*n* = 8). Significant differences between Mg-deficient roots and controls were made by unpaired *t*-test. Four means [two (Mg levels) × two (leaf positions)] were analyzed by two ANOVA followed by Duncan’s new multiple range test. Different letters above the bars indicate a significant difference at *P* < 0.05

We assayed the concentrations of GSH, GSSG, ASC and DHA, the important small molecular substances involved in the detoxification of ROS and MG, in the leaves and roots (Fig. 8). Mg-deficiency increased GSSG concentration, decreased GSH concentration and GSH/(GSH + GSSG) ratio in the leaves and roots, with a greater change in the lower leaves and roots than in the upper leaves. However, GSH + GSSG concentration was not significantly altered in the Mg-deficient leaves and roots. Compared with the Mg-deficient upper leaves, the Mg-deficient lower leaves had higher concentration of GSSG and lower ratio of GSH/(GSH + GSSG), but similar concentrations of GSH + GSSG and GSH. Under Mg-sufficiency, all the four parameters were similar between the lower and upper leaves (Fig. 8a-d and i-l).

As shown in Fig. 8e-h and m-p, the concentrations of ASC + DHA, ASC and DHA were greatly increased and decreased in the Mg-deficient lower leaves and roots, respectively, while the ratio of ASC/(ASC + DHA) was slightly decreased in the Mg-deficient lower leaves and roots. All the four parameters kept unchanged in the Mg-deficient upper leaves. Under Mg-deficiency, the lower leaves had higher concentrations of ASC + DHA, ASC and DHA and lower ratio of ASC/(ASC + DHA) relative to the upper leaves. Under Mg-sufficiency, all the four parameters did not significantly differ between the two.

In conclusions, Mg-deficiency affected antioxidants more in the lower leaves and roots than those in the upper leaves. The concentrations of antioxidants and the ratios of GSH/(GSH + GSSG) and ACS/(ASC + DHA) were significantly different between the Mg-deficient lower and upper leaves except for GSH and GSH + GSSG concentrations, but they were similar between the Mg-sufficient lower and upper leaves.

### MG in relation to GSH, Gly I and Gly II in leaves

**Fig. 9:**
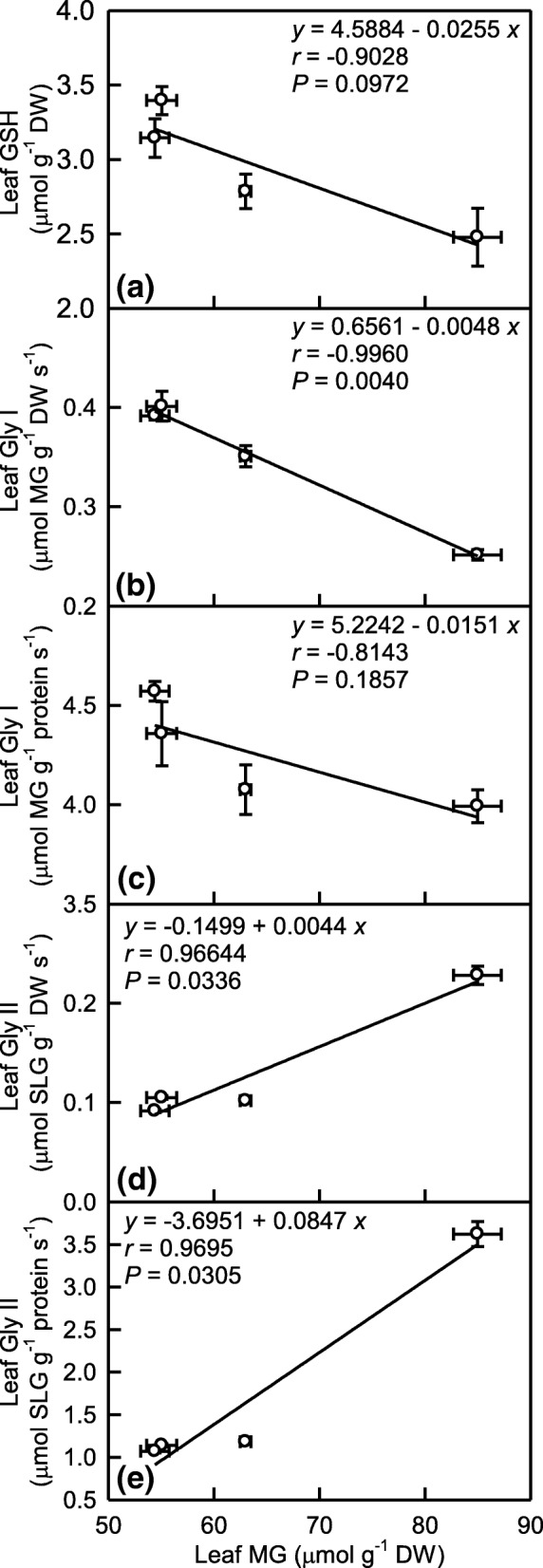
MG concentration in relation to GSH concentration (**a**), Gly I (**b-c**) and Gly II (**d-e**) activities expressed on a DW (**b** and **d**) or protein (**c** and **e**) basis in *C. sinensis* leaves. Data are from Figs. 3, 7 and 8. Points represent means ± SE (*n* = 8)

Here, we calculated the linear correlation coefficients between MG concentration and GSH concentration, Gly I or Gly II activity in order to investigate their roles in the detoxification of MG (Fig. 9). Leaf MG concentration displayed a significant and linear decrease with increasing Gly I activity on a DW basis and a decreased trend with increasing GSH concentration and Gly I activity on a protein basis. However, leaf MG concentration increased linearly and significantly with increasing Gly II activity, regardless of how the data were expressed.

### PCA loading plots and correlation

**Fig. 10:**
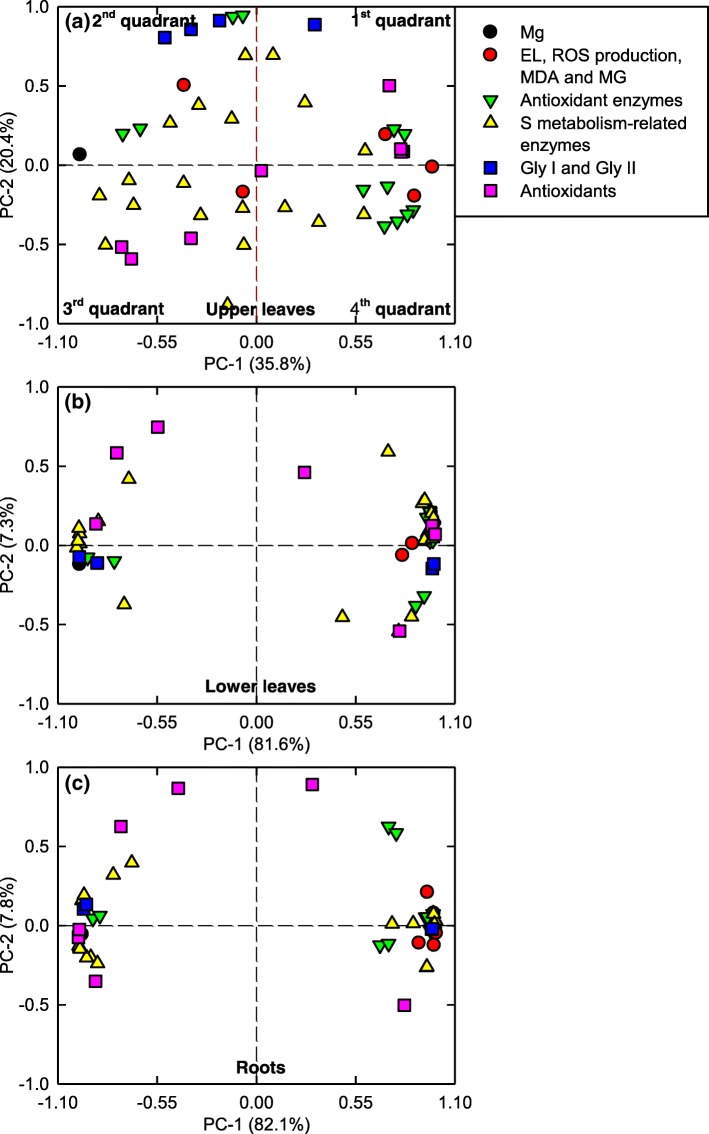
PCA loading plots of physiological parameter in *C. sinensis* upper leaves (**a**), lower leaves (**b**) and roots (**c**)

Through PCA, we revealed the differences in the response patterns of ROS and MG metabolisms to Mg-deficiency among the upper and lower leaves and roots (Fig. 10; Additional files 1: Tables S1, S2, S3). In the lower leaves and roots, most of ROS and MG metabolism-related parameters were highly clustered into left and right two groups. However, no obvious clustered parameters were observed in the upper leaves. The first two components comprised 88.9% (81.6% for PC1 and 7.3% for PC2) and 89.9% (82.1% for PC1 and 7.8% for PC2) of the total variation in the lower leaves and roots, respectively, but only 56.2% (PC1 for 35.8% and PC2 for 20.4%) in the upper leaves. Evidently, the Mg-deficient effects on these parameters were far less in the upper leaves and roots than those in the lower leaves. For the upper leaves, MDA (0.9766), Mg (− 0.9764), MG (0.8772), APR-D (−D: enzyme activity expressed on a DW basis; − 0.8695), APX-P (-P: enzyme activity expressed on a protein basis; 0.8692), DHAR-P (0.8362), SOD-P (0.8216) and ASC + DHA (0.8140) were the most influential in the PC1. For the lower leaves, the PC1 was loaded largely on CS-D (− 0.9960), DHA (0.9897), CS-P (− 0.9883), Gly II-P (0.9837), γGT-D (− 0.9831), DHAR-P (0.9820), γGCS-P (0.9815) and Gly I-D (− 0.9806). For the roots, PC1 was the mostly influenced by the alterations of MDA (0.9966), GST-P (0.9938), GST-D (0.9936), γGCS-P (0.9871), ASC (− 0.9858), ASC + DHA (− 0.9857), γGCS-D (0.9853) and electrolyte leakage (0.9849). It is worth noting that the Mg-deficiency-induced separation of these parameters also differed between the lower leaves and roots. For the lower leaves, seven parameters of antioxidants lay in the 1st and 2nd quadrants, only one parameter lay in the 4th quadrant; for the roots, three parameters of antioxidants lay in the 1st and 2nd quadrants, and five parameters lay in the 3rd and 4th quadrants.

**Fig. 11:**
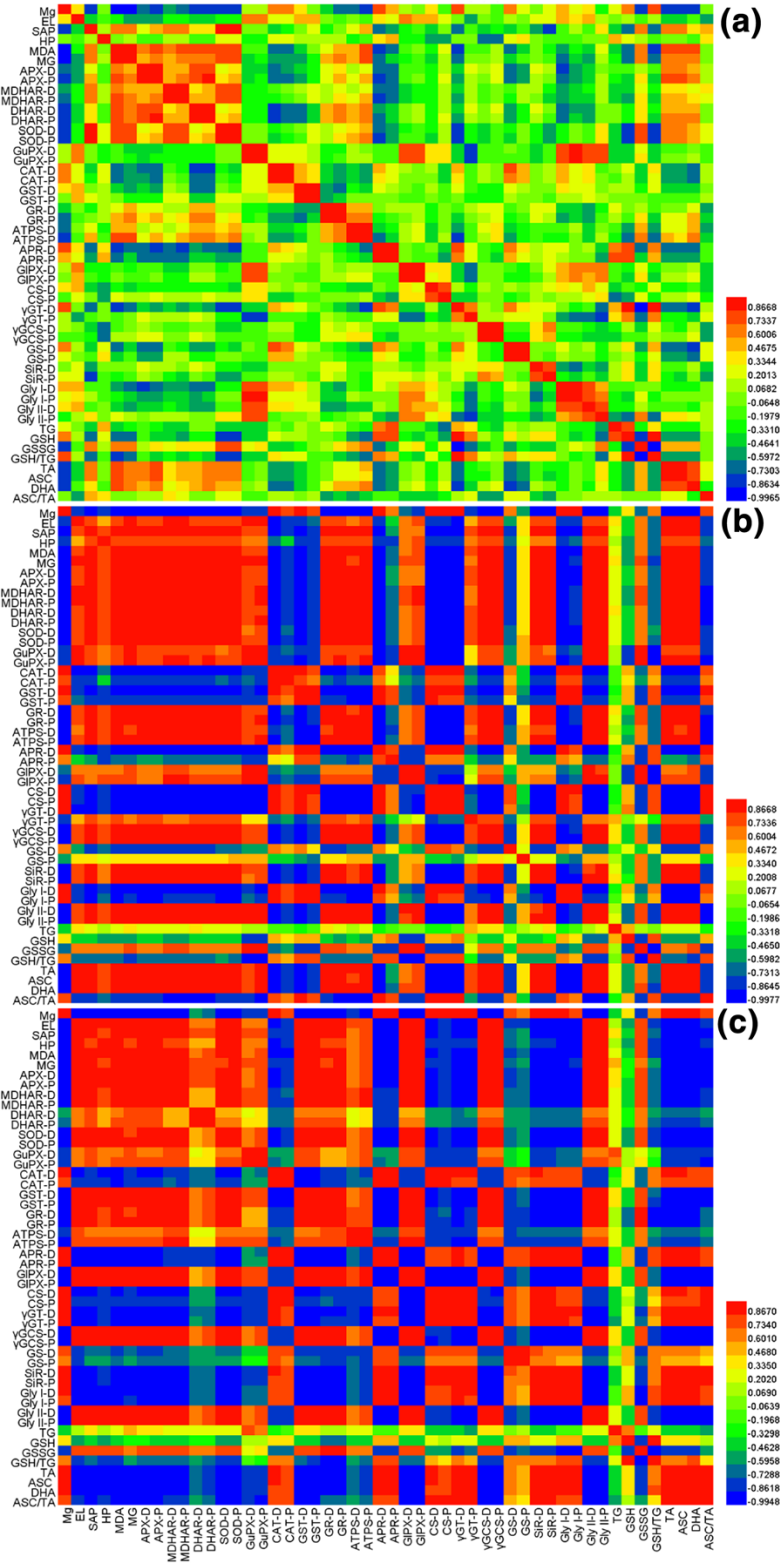
Matrices of Pearson correlation coefficients for 50 parameter in the upper (**a**) and lower (**b**) leaves and roots (**c**) of *C. sinensis* seedlings. EL: Electrolyte leakage; SAP, Superoxide anion production rate; HP: H_2_O_2_ production rate; −D: Enzyme activity expressed on a DW basis; -P: Enzyme activity expressed on a protein basis

Pearson correlation analysis was made using all the parameters for PCA in order to understand the relationships between these parameters (Fig. 11). Majority of these parameters were positively or negatively related with each other in the lower leaves and roots, but did not display any clear relationships in the upper leaves. Compared with the roots, more positive and less negative relationships between the parameters existed in the lower leaves.

## Discussion

### Responses of ROS and MG metabolisms to Mg-deficiency were far greater in the lower leaves and roots than those in the upper leaves, and Mg-deficiency enhanced greatly their differences between the lower and upper leaves

**Fig. 12:**
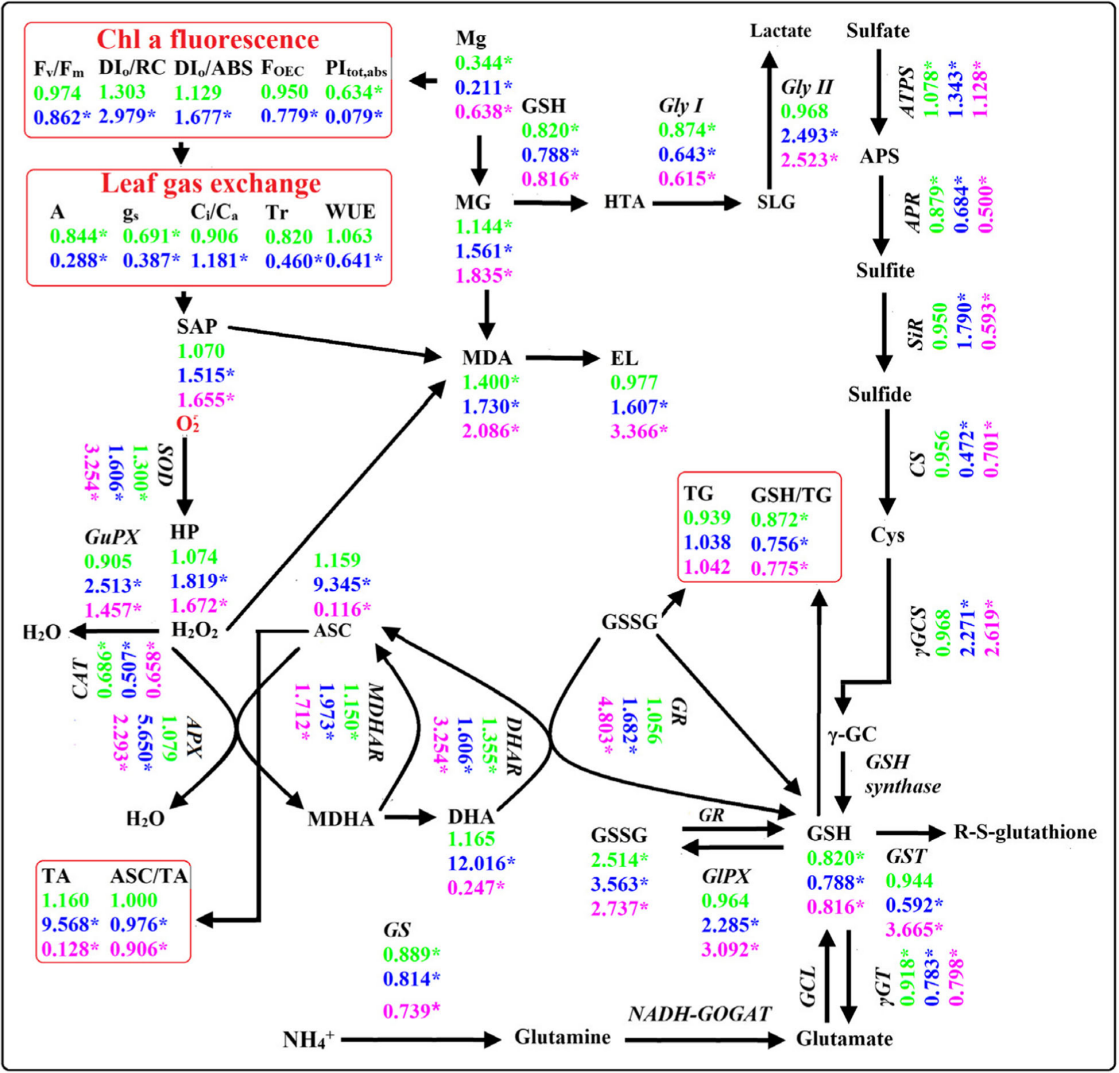
A diagram showing the Mg-deficient effects on gas exchange, Chl a fluorescence parameters, ROS and MG metabolism in *C. sinensis* roots (magenta), lower (in blue) and upper (in green). In this Figure, we used italics for enzymes and plain format for metabolites. Data from Figs.1–8. Enzyme activities were expressed on a DW basis. Values were the ratios of Mg-deficiency to Mg-sufficiency. An asterisk indicates a significant difference between Mg-deficiency and Mg-sufficiency at *P* < 0.05. An enzyme or metabolite was considered increased or decreased when it had both a relative change of more or less, respectively, than 1 and a *P*-value of < 0.05. Metabolite concentrations or enzyme activities were assayed on a whole tissue extract and not on a subcellular level. GCL: Glutamate-cysteine ligase; g_s_: Stomatal conductance; NADH-GOGAT: NADH-dependent glutamine-2-oxoglutarate aminotransferase; γGC: γ-glutamylcysteine; TA: ASC + DHA; TG: GSH + GSSG; Tr: transpiration

Based on our findings and the available data in the literatures, a scheme displayed the Mg-deficient effects on gas exchange, Chl a fluorescence, Mg, and ROS and MG metabolisms in the leaves and roots was presented here (Fig. 12). Under Mg-deficiency, all these parameters (60 parameters in the leaves and 50 parameters in the roots) were significantly altered in the lower leaves and roots except for GSH + GSSG concentration, but only 25 parameters were significantly altered in the upper leaves. Moreover, the Mg-deficiency-induced alterations of the 25 (22 parameters in roots) parameters were far less in the upper leaves than those in the lower leaves (roots) (Figs. 1-8). The only exception was that the Mg-deficiency-induced decrease in Mg level was greater in the upper leaves than that in the roots. Obviously, the responses of all these parameters to Mg-deficiency were far more pronounced in the lower leaves and roots than those in the upper leaves. It is worth mentioning that many (35) of parameters were not significantly affected in the Mg-deficient upper leaves. This may be related to the fact that the transport of Mg from older leaves to the younger leaves is improved under Mg-deficient conditions, thus providing Mg for the normal growth and development of the young leaves [38, 68], since Mg-deficiency-induced decrease was significantly less in the upper leaves than that in the lower leaves (Fig. 1a). This is also supported by our observation that the typical symptom of Mg-deficiency occurred only in the Mg-deficient lower leaves (Additional file 1: Fig. S1). In a study with *C. sinensis*, Li et al. [3] reported that many of physiological parameters (namely gas exchange, the concentrations of pigments, OA, total soluble proteins, amino acids and phenolics, and the activities of key enzymes involved in OA, amino acid and phenolic metabolisms) were significantly affected in the Mg-deficient lower leaves, but not in the Mg-deficient upper leaves. As shown in Fig. 10, all these parameters for PCA were highly clustered into the left and right two groups in the lower leaves and roots, but not in the upper leaves. The majority of parameters for PCA were positively or negatively related with each other only in the lower leaves and roots, but not in the upper leaves (Fig. 11). Obviously, the responses of ROS and MG metabolisms to Mg-deficiency occurred highly only in the lower leaves and roots rather than in the upper leaves. In a word, the Mg-deficient effects on ROS and MG metabolisms were far greater in the lower leaves and roots than those in the upper leaves.

As shown in Figs. 3-8, many of ROS and MG metabolism-related parameters differed significantly only between the Mg-deficient lower and upper leaves, but not between the Mg-sufficient lower and upper leaves. Although leaf positions had influence on some of parameters under Mg-sufficiency, the influence was far less than under Mg-deficiency. Evidently, the differences in ROS and MG metabolisms between the lower and upper leaves were greatly elevated by Mg-deficiency.

### ROS detoxification systems as a whole did not provide considerable protect from oxidative damage in the Mg-deficient leaves and roots

Nutrient deficiencies can break the balance between the production of ROS and their removal via detoxification systems, thus causing ROS over-accumulation and lipid peroxidation in root and leaf cells [1, 2, 16, 24, 69, 70]. Here, the production rates of superoxide anion and H_2_O_2_ and the concentration of MDA were increased in the Mg-deficient leaves and roots, with a greater increase in the Mg-deficient lower leaves and roots than those in the upper leaves except for leaf H_2_O_2_ generation rate (Fig. 3b-d and g-i). Regressive analysis showed that leaf superoxide anion or H_2_O_2_ production rate increased linearly with increasing DI_o_/RC or DI_o_/ABS, and decreased linearly with increasing CO_2_ assimilation, F_v_/F_m_, the fraction of OEC in comparison with control or PI_tot,abs_ (Table 1). Thus, the greater increases in the ROS production rates in the Mg-deficient lower leaves might be caused by more excess absorbed light energy due to decreased photosynthetic electron transport resulting from a larger decrease in CO_2_ assimilation. It is worth noting that leaf MDA concentration increased linearly with increasing MG concentration, but only displayed an upward trend with increasing superoxide anion or H_2_O_2_ production rate (Table 1). Over-accumulation of MG can also lead to lipid peroxidation in plant cells [35, 36]. Yadav et al. [36] reported that the salt-induced increases of MG and MDA concentrations in the leaves were much less in *Gly I* and/or *Gly II* overexpressing transgenic tobacco plants than those in wild-type (WT) plants. Thus, it is reasonable to assume that in addition to increased ROS production rate, the more pronounced lipid peroxidation in the Mg-deficient lower leaves could be explained by the larger increase in the MG concentration (Fig. 2). Regressive analysis indicated that leaf electrolyte leakage only displayed an increased trend with increasing MDA concentration, but increased linearly with increasing MG concentration (Table 1). MG is a potent reactive cytotoxin which can disrupt biomembrane structures and functions [35]. Kumar et al. [71] reported that transgenic tobacco plants overexpressing *ALDRXV4* encoding an aldose reductase, which is involved in the convert of MG into acetol, had enhanced tolerance to drought and salinity by scavenging MG and lowering electrolyte leakage. Thus, the difference in the electrolyte leakage between the Mg-deficient lower and upper leaves could be at least partially explained by the larger increase in the MG level in the Mg-deficient lower leaves than that in the Mg-deficient upper leaves (Fig. 2).

Plants have evolved efficient enzymatic and non-enzymatic detoxification systems of ROS to protect plant cells from oxidative damage [18–20, 65]. In this study, the Mg-deficiency-induced increases in the activities of APX, MDHAR, DHAR, SOD and GuPX were greater in the Mg-deficient lower leaves and roots than those in the upper leaves (Fig. 4). The larger increases of antioxidant enzyme activities in the Mg-deficient lower leaves and roots than those in the Mg-deficient upper leaves agreed with the increased requirement for the removal of ROS (Fig. 3). The Mg-deficiency-induced upregulation of antioxidant enzymes have also been reported on many higher plants as mentioned in the background. It is a remarkable fact that among the six antioxidant enzymes, only the activity of CAT was decreased in the Mg-deficient leaves and roots (Fig. 4f, l, r and x). Similar results have been obtained on the N-deficient grape leaves [18], B-stressed and Mg-deficient *Citrus* leaves [2, 14, 67, 72]. The larger decrease in CAT activity in the Mg-deficient lower leaves and roots than that in the Mg-deficient upper leaves might be related to the fact that CAT is very sensitive to oxidative stress [73, 74], and that the protein level of CAT can be decreased rapidly under conditions that inhibit translation such as salt, cold, high light, heat-shock or senescence [74–76], because the production rates of superoxide anion and H_2_O_2_were greatly increased in the Mg-deficient lower leaves and roots, but slightly increased and significantly unaltered in the Mg-deficient upper leaves, respectively (Fig. 3).

As shown in Figs. 5-6, the activities of all the ten enzymes involved in S metabolism were significantly altered in the Mg-deficient lower leaves and roots, but kept unchanged in the Mg-deficient upper leaves except for slightly increased activity of ATPS on a DW or protein basis, and slightly decreased activities of APR on a DW or protein basis and of γGT and GS on a DW basis. Similarly, the Mg-deficiency-induced alterations of (GSH + GSSG), GSH and GSSG concentrations and GSH/(GSH + GSSG) ratio were greater in the lower leaves and roots than those in the upper leaves (Fig. 8a-d and i-l). Obviously, the Mg-deficiency affected the thiol-based ROS detoxification system more in the lower leaves and roots than that in the upper leaves. This agreed with our data that the Mg-deficiency-induced of increases in ROS production rates and MDA accumulation were greater in the lower leaves and roots than those in the upper leaves (Fig. 3). The level of GSH in a given plant cell is the result of the coordinated actions of GSH biosynthesis, utilization and degradation [65]. CS converts sulfide to Cys, a key factor for GSH biosynthesis [77]. The biosynthesis of GSH from Cys is catalyzed by γGCS (a rate-limiting enzyme) and glutathione synthetase [78]. GS also participates in GSH biosynthesis via glutamate biosynthesis pathway [79]. The GSH formed can be utilized in diverse redox reactions to protect the plant cells against oxidative stress, thus leading to the oxidation of GSH to GSSG, which is reduced to GSH by GR [65]. For example, GlPX catalyzes mainly the conversion of H_2_O_2_ to H_2_O at the expense of GSH, thereby producing GSSG [20, 78]. GSTs catalyze the conjugation of a range of toxicants or their metabolites to GSH and thus lower their toxicity. Many GSTs have been shown to possess GlPX activity [65, 80]. Evidence shows that γGT plays a key role in the degradation of GSH [81]. Therefore, the Mg-deficiency-induced decrease in GSH level might be caused by decreased biosynthesis due to decreased DW-based GS and/or CS activities and/or by the increased utilization due to increased DW-based GlPX activity (GST and GlPX activities) in the leaves (roots); the Mg-deficiency-induced increase of GSSG level might be caused by increased oxidation of GSH to GSSG due to increased activity of GlPX (GST and GlPX activities) in the lower leaves (roots) rather than by decreased reduction of GSSG to GSH by GR, whose activity was increased under Mg-deficiency. However, the activities of GST, GR and GlPX were not significantly altered in the Mg-deficient upper leaves, the Mg-deficiency-induced increase of GSSG level in these leaves should be caused by other factors (Figs. 5-6). It is worth noting that MG is spontaneously converted to HTA using one molecule of GSH, that the resultant HTA is then catalyzed by Gly I and Gly II to lactate and one molecule of GSH is recycled back into the Gly system, and that the Gly system is involved in the maintenance of GSH homeostasis [34, 35]. Thus, the Mg-deficiency-induced larger alterations of MG level and Gly system (Figs. 3e, j and 7) might also contribute to Mg-deficiency-induced larger decrease in GSH concentration in the lower leaves and roots than that in the upper leaves.

Although the activities of most antioxidant and S metabolism-related enzymes and the concentration of GSH in the Mg-deficient leaves and roots and the concentration of ASC in the Mg-deficient leaves were kept in higher levels (Figs. 4-6 and 8), MDA concentration and/or electrolyte leakage were increased in the Mg-deficient lower and upper leaves and roots (Fig. 3a, d, f and i). Therefore, the ROS detoxification systems as a whole did not protect effectively these Mg-deficient leaves and roots from oxidative damage. This is also supported by our finding that the ratio of GSH to GSH + GSSG, which decreases under oxidative stress [82, 83], was decreased in the Mg-deficient lower and upper leaves and roots (Fig. 8d and l).

### Possible causes for the Mg-deficiency-induced accumulation of MG in the leaves and roots

The major route for the detoxification of MG is through the coordinated actions of Gly I and Gly II using GSH as a cofactor. GSH availability is very essential for the detoxification of MG [34, 35]. Our results showed that the Mg-deficient lower leaves and roots had decreased activity of Gly I and increased activity of Gly II, but their activities remained unchanged in the Mg-deficient upper leaves except for a slight decrease in the DW-based Gly I activity (Fig. 7), and that the Mg-deficiency-induced decrease of GSH concentration was greater in the lower leaves and roots than that in the upper leaves (Fig. 8b and j). As shown in Fig. 9, leaf MG concentration increased linearly with decreasing DW-based Gly I activity, and had an increased trend with decreasing protein-based Gly I activity or GSH level; but increased linearly with increasing Gly II activity. Thus, the Mg-deficiency-induced increase of MG level in the Mg-deficient leaves and roots might be caused by the Mg-stimulated production of MG and the decreased MG detoxification capacity due to decreased GSH concentration (Fig. 8e and j) and Gly I activity on a DW and/or protein basis (Fig. 7a, c, e and g). In a word, the Gly system as a whole did not provide sufficient MG detoxification capacity to prevent the Mg-deficiency-induced production and accumulation of MG in the lower and upper leaves and roots.

### Some differences existed in the Mg-deficiency-induced alterations of ROS and MG metabolisms between the leaves and roots

As shown in Fig. 12, Mg-deficiency affected gas exchange, Chl a fluorescence, and ROS and MG metabolisms more in the lower leaves than those in the upper leaves, but they displayed similar change patterns between Mg-deficient lower and upper leaves. By contrast, most of ROS and MG metabolism-related parameters had similar variation degrees and patterns between the Mg-deficient lower leaves and roots, but several parameters showed the opposite change trends. For example, the Mg-deficient lower leaves had a decreased GST activity and an increased SiR activity, but the reverse was this case for the Mg-deficient roots (Figs. 5-6). In addition, the concentrations of ASC + DHA, ASC and DHA were greatly increased in the Mg-deficient lower leaves (Fig. 8e-g), as previously obtained on the Mg-deficient *Citrus* leaves [2], but their concentrations were greatly reduced to low levels in the Mg-deficient roots (Fig. 8m-o). PCA showed that the distributions of some parameters in the four quadrants were different (Fig. 10). Obviously, some differences existed in the Mg-deficiency-induced alterations of ROS and MG metabolisms between the lower leaves and roots. This is also supported by the different PCA correlation coefficients for parameters related to ROS and MG metabolisms in the lower leaves and roots (Fig. 11).

## Conclusions

The Mg-deficiency-induced alterations of Mg level, gas exchange, Chl a fluorescence, and ROS and MG metabolisms were more pronounced in the lower leaves than those in the upper leave, but all the measured parameters showed similar change patterns between the Mg-deficient lower and upper leaves. This may be related to the fact that the transport of Mg from old leaves to the young leaves is improved under Mg-deficient conditions in order to provide Mg for the normal growth and development of the young leaves. Accordingly, their differences between the lower and upper leaves were greatly intensified by Mg-deficiency. Most of ROS and MG metabolism-related parameters had similar variation degrees and trends between the Mg-deficient lower leaves and roots, but several parameters (i.e., GST, SiR, ASC + DHA, ASC and DHA) had the opposite variation trends. Obviously, some differences existed in the Mg-deficiency-induced alterations of ROS and MG metabolisms between the lower leaves and roots. The ROS and MG detoxification systems as a whole did not provide sufficient ROS and MG detoxification capacity to prevent the Mg-deficiency-induced production and accumulation of ROS and MG, thus leading to lipid peroxidation and the loss of plasma membrane integrity (increase of electrolyte leakage), especially in the lower leaves and roots.

## Additional files

Additional file 1: **Figure S1.** Mg-deficiency effects on *Citrus sinensis* seedling growth (**a**) and Mg-deficient symptoms in the upper and lower leaves (**b**). **Table S1.** PCA for physiological parameters of upper leaves. **Table S2.** PCA for physiological parameters of lower leaves. **Table S3.** PCA for physiological parameters of roots. (DOCX 447 kb)

## Abbreviations

APR: Adenosine 5′-phosphosulfate reductase
APX: Ascorbate peroxidase
ASC: Ascorbate
ATPS: ATP sulphurylase
CAT: Catalase
Chl: Chlorophyll
CS: Cysteine synthase
Cys: Cysteine
DHA: Dehydroascorbate
DHAR: DHA reductase
DI_o_/ABS: Quantum yield for energy dissipation
DI_o_/RC: Specific energy fluxes per reaction center for energy dissipation
DTT: Dithiothreitol
F_v_/F_m_: Maximum PSII efficiency of dark-adapted leaves
GlPX: Glutathione peroxidase
Gly I: Glyoxalase I
Gly II: Glyoxalase II
GR: Glutathione reductase
GS: Glutamine synthetase
GSH: Reduced glutathione
GSSG: Oxidized glutathione
GST: Glutathione S-transferase
GuPX: Guaiacol peroxidase
MDA: Malondialdehyde
MDHA: Monodehydroascorbate
MDHAR: MDHA reductase
Mg: Magnesium
MG: Methylglyoxal
OA: Organic acid
OAS: O-acetyl-l-serine
OEC: Oxygen-evolving complex
OJIP transient: Chl a fluorescence transient
PI_tot,abs_: Total performance index
PSII: Photosystem II
RC: Reaction center
ROS: Reactive oxygen species
SiR: Sulfite reductase
SLG: S-D-lactoylglutathione
SOD: Superoxide dismutase
TCA: Trichloroacetic acid
WUE: Water use efficiency
γGCS: γ-glutamylcysteine synthase
γGT: γ-glutamyltransferase
