# Supplementary material for: Responses of reactive oxygen species and methylglyoxal metabolisms to magnesium-deficiency differ greatly among the roots, upper and lower leaves of Citrus sinensis
Source: BMC Plant Biol. 2019 Feb 15;19:76. doi: 10.1186/s12870-019-1683-4 (PMC6377732; doi:10.1186/s12870-019-1683-4)
Supplement: Supplementary file 1 — Figure S1. Mg-deficiency effects on Citrus sinensis seedling growth (a) and Mg-deficient symptoms in the upper and lower leaves (b). Table S1. PCA for physiological parameters of upper leaves. Table S2. PCA for physiological parameters of lower leaves. Table S3. PCA for physiological parameters of roots. (DOCX 447 kb) [file 12870_2019_1683_MOESM1_ESM.docx]

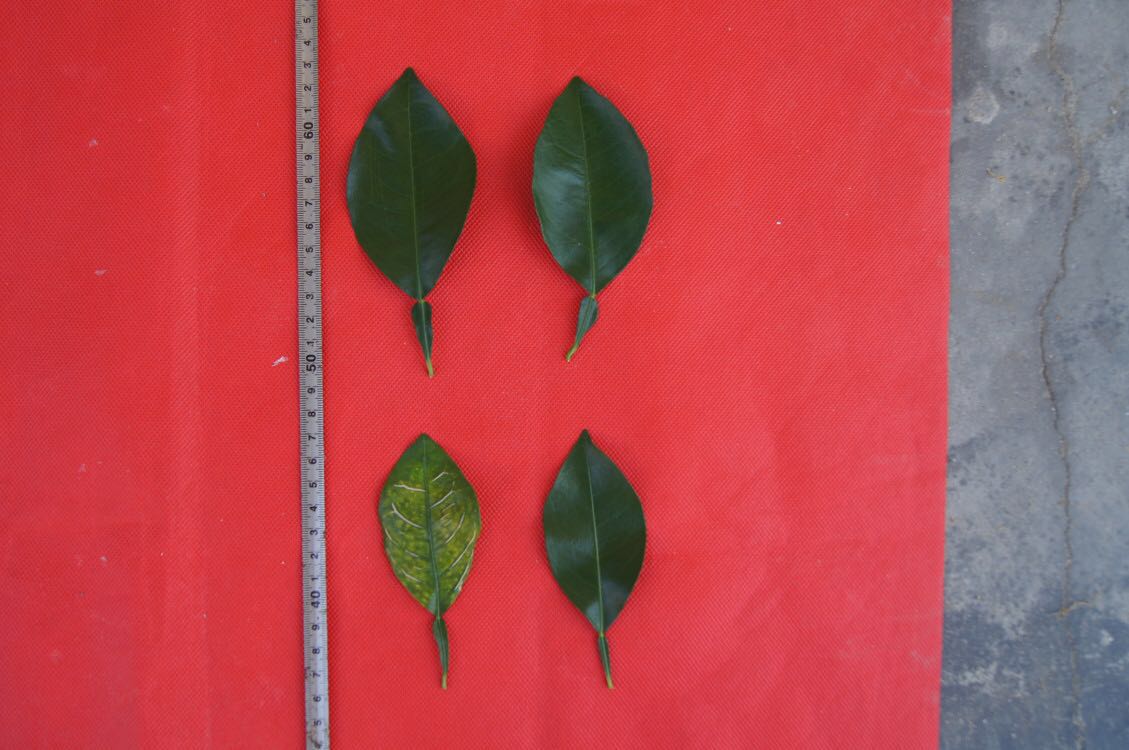

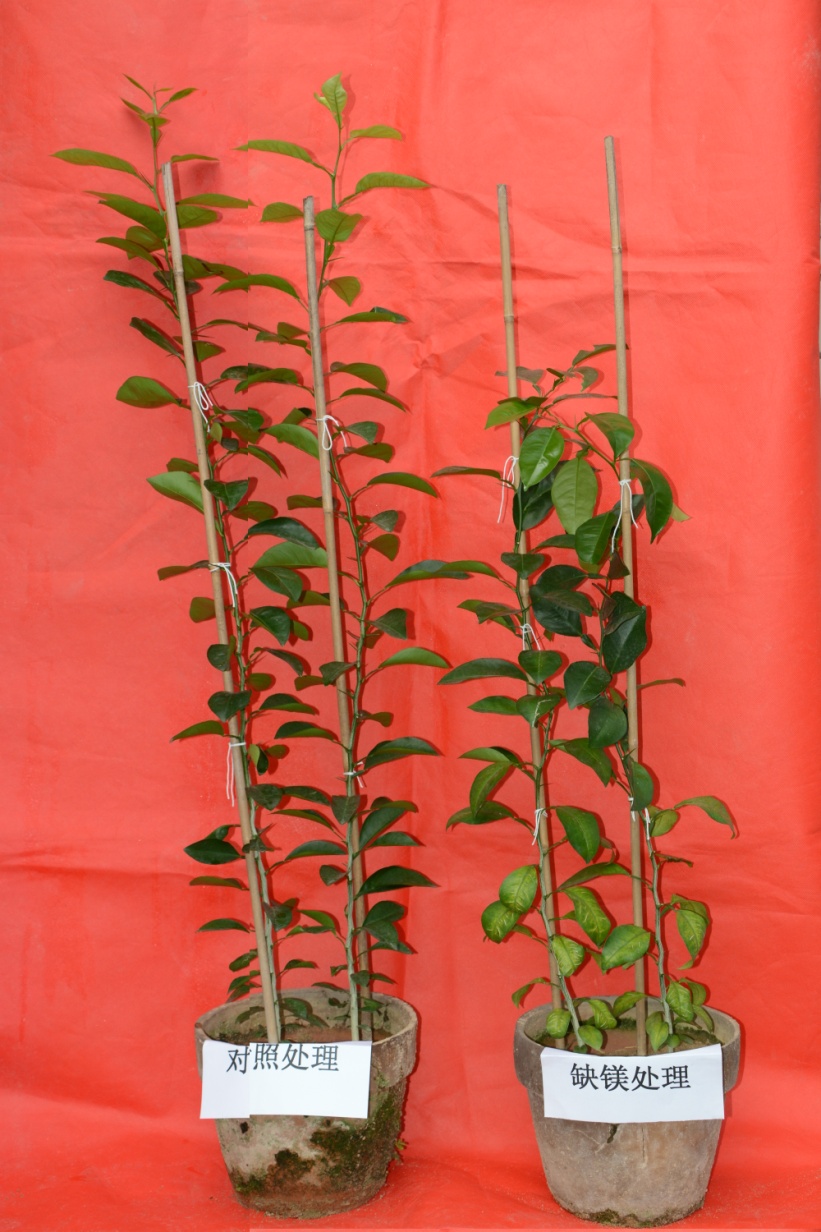


**b**

**a**

**Control**

**Mg-deficiency**

**Lower leaves Upper leaves**

**Mg-deficiency**

**Control**

**Additional file 1: Figure S1.** Mg-deficiency effects on *Citrussinensis* seedling growth (**a**) and Mg-deficient symptoms in the upper and lower leaves (**b**).

**Additional file 2: Table S1.** PCA for physiological parameters of upper leaves. -D, enzyme activity expressed on a dry weight (DW) basis; -P, enzyme activity expressed on a protein basis.

| **Variables** | **PC1** | **PC2** | **PC3** | **PC4** | **PC5** | **PC6** | **PC7** | **PC8** | **PC9** |
| --- | --- | --- | --- | --- | --- | --- | --- | --- | --- |
| **Mg** | -0.9764 | 0.0640 | 0.0585 | -0.1264 | -0.0826 | -0.0322 | 0.0367 | 0.0461 | -0.0522 |
|  |  |  |  |  |  |  |  |  |  |
| **Electrolyte leakage, ROS production rates, MDA and MG** | | | |  |  |  |  |  |  |
| Electrolyte leakage | -0.4002 | 0.5023 | 0.3515 | 0.0948 | 0.2824 | 0.5415 | 0.0249 | -0.1891 | 0.1315 |
| Superoxide anion production rate | 0.7166 | 0.1918 | -0.6181 | -0.0618 | -0.0091 | -0.0831 | -0.1449 | -0.1350 | 0.0940 |
| H_2_O_2_ production rate | -0.0722 | -0.1719 | -0.5987 | 0.6075 | 0.0664 | 0.2721 | 0.1919 | 0.2629 | -0.2219 |
| MDA | 0.9766 | -0.0136 | -0.0269 | -0.0416 | 0.0750 | 0.1226 | 0.0190 | -0.0785 | -0.0050 |
| MG | 0.8772 | -0.1963 | -0.0830 | 0.1364 | 0.2370 | -0.1313 | 0.1178 | 0.0656 | -0.2616 |
|  |  |  |  |  |  |  |  |  |  |
| **Antioxidant enzymes** |  |  |  |  |  |  |  |  |  |
| APX-D | 0.7096 | -0.3846 | 0.1544 | -0.2852 | -0.3020 | 0.1908 | 0.0514 | 0.2237 | 0.1636 |
| APX-P | 0.8692 | -0.2838 | 0.0968 | -0.1960 | -0.2182 | 0.1179 | 0.0320 | 0.1401 | 0.1338 |
| MDHAR-D | 0.5927 | -0.1550 | -0.4758 | -0.3495 | 0.3076 | 0.2215 | 0.2015 | 0.1037 | 0.2230 |
| MDHAR-P | 0.7267 | -0.1341 | -0.4236 | -0.2918 | 0.2706 | 0.1782 | 0.1664 | 0.0723 | 0.1862 |
| DHAR-D | 0.7811 | -0.3553 | 0.2299 | 0.1201 | -0.0869 | 0.0424 | 0.2307 | 0.0664 | 0.3402 |
| DHAR-P | 0.8362 | -0.3118 | 0.1939 | 0.1076 | -0.0840 | 0.0260 | 0.2005 | 0.0382 | 0.3008 |
| SOD-D | 0.7628 | 0.2266 | -0.4965 | -0.0017 | 0.2771 | 0.0637 | 0.0399 | -0.0392 | -0.1397 |
| SOD-P | 0.8216 | 0.1969 | -0.4414 | 0.0056 | 0.2481 | 0.0568 | 0.0286 | -0.0359 | -0.1223 |
| GuPX-D | -0.1308 | 0.9375 | 0.2304 | 0.0325 | 0.0650 | 0.0219 | 0.0351 | 0.1085 | 0.1790 |
| GuPX-P | -0.0757 | 0.9456 | 0.2277 | 0.0538 | 0.0640 | 0.0125 | 0.0230 | 0.1168 | 0.1640 |
| CAT-D | -0.7408 | 0.1987 | -0.2698 | -0.1582 | 0.3844 | 0.0004 | -0.0249 | 0.4038 | 0.0287 |
| CAT-P | -0.6440 | 0.2317 | -0.3242 | -0.1714 | 0.4435 | -0.0026 | -0.0369 | 0.4440 | 0.0237 |
|  |  |  |  |  |  |  |  |  |  |
| **S metabolism-related enzymes** | |  |  |  |  |  |  |  |  |
| GST-D | -0.4782 | 0.2658 | -0.3982 | -0.1982 | 0.2576 | 0.5574 | -0.2913 | -0.1562 | 0.1166 |
| GST-P | -0.1372 | 0.2918 | -0.4636 | -0.1907 | 0.3181 | 0.6052 | -0.3372 | -0.2018 | 0.1197 |
| GR-D | 0.3458 | -0.3607 | 0.4536 | 0.4635 | 0.3806 | -0.0242 | 0.2882 | 0.0391 | 0.0390 |
| GR-P | 0.5957 | -0.3113 | 0.3847 | 0.4179 | 0.3313 | -0.0330 | 0.2387 | 0.0101 | 0.0443 |
| ATPS-D | 0.6028 | 0.0909 | 0.3138 | 0.3159 | 0.5216 | -0.1602 | -0.2610 | -0.1614 | 0.1751 |
| ATPS-P | 0.8036 | 0.0722 | 0.2065 | 0.2431 | 0.3761 | -0.1223 | -0.2131 | -0.1436 | 0.1287 |
| APR-D | -0.8695 | -0.1922 | 0.0090 | 0.4282 | -0.0231 | 0.0703 | 0.0002 | -0.0393 | -0.0961 |
| APR-P | -0.6803 | -0.2536 | -0.0074 | 0.6435 | -0.0268 | 0.0970 | -0.0187 | -0.1000 | -0.1301 |
| GlPX-D | -0.0609 | 0.6921 | 0.5537 | 0.2337 | -0.0556 | 0.1089 | 0.1647 | 0.2431 | 0.2021 |
| GlPX-P | 0.0923 | 0.6943 | 0.5441 | 0.2609 | -0.0671 | 0.0917 | 0.1505 | 0.2340 | 0.1899 |
| CS-D | -0.3198 | 0.3781 | 0.3944 | -0.4544 | -0.1352 | 0.0929 | 0.4837 | -0.3342 | -0.1195 |
| CS-P | 0.2687 | 0.3942 | 0.3819 | -0.4334 | -0.1367 | 0.0766 | 0.4956 | -0.3889 | -0.1070 |
| γGT-D | -0.8363 | -0.5034 | -0.0443 | -0.1052 | -0.0547 | -0.0846 | 0.0251 | -0.0368 | 0.1352 |
| γGT-P | -0.1599 | -0.8838 | -0.1344 | -0.1299 | -0.0685 | -0.1845 | 0.0072 | -0.1639 | 0.2782 |
| γGCS-D | -0.0757 | -0.2716 | 0.6464 | -0.4055 | 0.3421 | -0.2693 | -0.3000 | 0.2328 | -0.0581 |
| γGCS-P | 0.1576 | -0.2672 | 0.6313 | -0.3941 | 0.3409 | -0.2842 | -0.3269 | 0.2075 | -0.0375 |
| GS-DW | -0.7059 | -0.0966 | -0.3441 | -0.0078 | 0.3635 | -0.2037 | 0.4205 | 0.0621 | 0.0113 |
| GS-P | -0.4031 | -0.1147 | -0.4704 | 0.0074 | 0.4669 | -0.2535 | 0.5248 | 0.0468 | 0.0208 |
| SiR-D | -0.0700 | -0.5041 | 0.4078 | -0.1834 | 0.2012 | 0.5780 | 0.2074 | 0.1472 | -0.2188 |
| SiR-P | -0.3093 | -0.3169 | 0.6665 | -0.1319 | 0.1901 | 0.3517 | 0.0397 | 0.3008 | -0.2114 |
|  |  |  |  |  |  |  |  |  |  |
| **Gly I and Gly II** |  |  |  |  |  |  |  |  |  |
| Gly I-D | -0.5069 | 0.8051 | -0.0012 | 0.0748 | -0.0139 | -0.2069 | -0.0976 | -0.0091 | 0.0917 |
| Gly I-P | -0.2035 | 0.9117 | -0.0133 | 0.1198 | -0.0081 | -0.2421 | -0.1335 | -0.0159 | 0.0930 |
| Gly II-D | -0.3630 | 0.8578 | -0.0483 | -0.0561 | -0.2271 | 0.0490 | 0.1625 | 0.1496 | -0.0119 |
| Gly II-P | 0.3235 | 0.8869 | -0.0867 | 0.0011 | -0.2145 | 0.0207 | 0.1434 | 0.1271 | -0.0133 |
|  |  |  |  |  |  |  |  |  |  |
| **Antioxidants** |  |  |  |  |  |  |  |  |  |
| GSH + GSSG | -0.3626 | -0.4625 | -0.0505 | 0.6850 | -0.2402 | 0.2788 | -0.0934 | 0.0149 | 0.1780 |
| GSH | -0.6932 | -0.5926 | 0.0571 | 0.1753 | -0.2066 | 0.1916 | -0.0563 | 0.0473 | 0.1941 |
| GSSG | 0.7362 | 0.5006 | -0.1286 | 0.3147 | 0.1079 | -0.0527 | 0.0059 | -0.0585 | -0.1415 |
| GSH/(GSH + GSSG) | -0.7468 | -0.5179 | 0.1361 | -0.2570 | -0.1330 | 0.0688 | 0.0030 | 0.0322 | 0.1529 |
| ASC + DHA | 0.8140 | 0.0853 | -0.0923 | -0.0044 | -0.3593 | 0.1104 | -0.1182 | 0.2449 | -0.1348 |
| ASC | 0.8034 | 0.0825 | -0.1324 | -0.0086 | -0.3743 | 0.0997 | -0.1076 | 0.2574 | -0.1260 |
| DHA | 0.7996 | 0.1014 | 0.3332 | 0.0390 | -0.1505 | 0.2036 | -0.2097 | 0.0791 | -0.2045 |
| ASC/(ASC + DHA) | 0.0264 | -0.0353 | -0.8157 | -0.0510 | -0.4048 | -0.1511 | 0.1087 | 0.3072 | 0.1371 |
|  |  |  |  |  |  |  |  |  |  |
| **Eigen value** | 17.8944 | 10.2366 | 6.5415 | 3.6593 | 3.1976 | 2.3879 | 2.0893 | 1.6336 | 1.2024 |
| **Variation percent (%)** | 35.7888 | 20.4732 | 13.0829 | 7.3186 | 6.3951 | 4.7758 | 4.1787 | 3.2672 | 2.4048 |

**Additional file 3: Table S2.** PCA for physiological parameters of lower leaves. -D, enzyme activity expressed on a dry weight (DW) basis; -P, enzyme activity expressed on a protein basis.

| **Variables** | **PC1** | **PC2** | **PC3** | **PC4** | **PC5** |
| --- | --- | --- | --- | --- | --- |
| **Mg** | -0.9787 | -0.1217 | -0.0756 | 0.0302 | -0.0308 |
|  |  |  |  |  |  |
| **Electrolyte leakage, ROS production rates, MDA and MG** | | |  |  |  |
| Electrolyte leakage | 0.8656 | 0.0113 | -0.2261 | -0.3030 | 0.2355 |
| Superoxide anion production rate | 0.9683 | 0.2065 | 0.0221 | -0.0294 | -0.0781 |
| H_2_O_2_ production rate | 0.8098 | -0.0644 | 0.2377 | 0.4395 | -0.0694 |
| MDA | 0.9772 | 0.0982 | -0.0127 | 0.0983 | -0.0847 |
| MG | 0.9682 | 0.0241 | -0.0585 | 0.0456 | 0.1443 |
|  |  |  |  |  |  |
| **Antioxidant enzymes** |  |  |  |  |  |
| APX-D | 0.9577 | 0.2186 | -0.0356 | -0.0175 | -0.0633 |
| APX-P | 0.9613 | 0.2108 | -0.0327 | -0.0175 | -0.0615 |
| MDHAR-D | 0.9619 | 0.0274 | -0.1151 | 0.0074 | 0.1290 |
| MDHAR-P | 0.9781 | 0.0315 | -0.0966 | 0.0063 | 0.0848 |
| DHAR-D | 0.9599 | 0.1477 | -0.0176 | 0.0376 | 0.0786 |
| DHAR-P | 0.9820 | 0.1134 | -0.0234 | 0.0146 | 0.0334 |
| SOD-D | 0.9450 | 0.1747 | 0.1289 | -0.0003 | -0.1385 |
| SOD-P | 0.9723 | 0.1341 | 0.0887 | -0.0018 | -0.0997 |
| GuPX-D | 0.8831 | -0.3818 | -0.1004 | -0.0990 | 0.1245 |
| GuPX-P | 0.9301 | -0.3198 | -0.0736 | -0.0718 | 0.0854 |
| CAT-D | -0.9353 | -0.0768 | 0.1803 | 0.2527 | 0.0619 |
| CAT-P | -0.7873 | -0.0998 | 0.3410 | 0.4475 | 0.0485 |
|  |  |  |  |  |  |
| **S metabolism-related enzymes** |  |  |  |  |  |
| GST-D | -0.9785 | 0.0111 | -0.1392 | 0.0164 | 0.0983 |
| GST-P | -0.8762 | 0.1511 | -0.3342 | 0.0409 | 0.1853 |
| GR-D | 0.9484 | 0.0792 | 0.0224 | 0.0551 | 0.0859 |
| GR-P | 0.9787 | 0.0807 | 0.0150 | 0.0428 | 0.0296 |
| ATPS-D | 0.9307 | 0.0330 | 0.1657 | 0.0656 | -0.1749 |
| ATPS-P | 0.9756 | 0.0423 | 0.0998 | 0.0278 | -0.1105 |
| APR-D | -0.9801 | 0.0741 | -0.1374 | -0.0980 | -0.0019 |
| APR-P | -0.7075 | 0.4170 | -0.4446 | -0.3129 | -0.0534 |
| GlPX-D | 0.7879 | -0.5450 | -0.1395 | 0.0933 | 0.1372 |
| GlPX-P | 0.8594 | -0.4507 | -0.0975 | 0.0887 | 0.1126 |
| CS-D | -0.9960 | -0.0156 | 0.0388 | -0.0110 | 0.0288 |
| CS-P | -0.9883 | 0.0280 | 0.0752 | -0.0059 | 0.0121 |
| γGT-D | -0.9831 | 0.1088 | 0.0838 | 0.0376 | 0.0192 |
| γGT-P | 0.7303 | 0.5899 | 0.2457 | 0.0993 | -0.1050 |
| γGCS-D | 0.9706 | 0.2036 | 0.0487 | 0.0501 | 0.0447 |
| γGCS-P | 0.9815 | 0.1714 | 0.0277 | 0.0345 | 0.0131 |
| GS-D | -0.7322 | -0.3738 | 0.3764 | -0.4170 | 0.0084 |
| GS-P | 0.4765 | -0.4541 | 0.5152 | -0.5283 | -0.1029 |
| SiR-D | 0.9202 | 0.2660 | -0.1202 | 0.0145 | 0.0160 |
| SiR-P | 0.9325 | 0.2848 | -0.0413 | -0.0130 | -0.0150 |
|  |  |  |  |  |  |
| **Gly I and Gly II** |  |  |  |  |  |
| Gly I-D | -0.9806 | -0.0739 | -0.0518 | 0.0860 | 0.1311 |
| Gly I-P | -0.8825 | -0.1112 | -0.1748 | 0.2382 | 0.3008 |
| Gly II-D | 0.9755 | -0.1470 | -0.0754 | 0.0203 | 0.0411 |
| Gly II-P | 0.9837 | -0.1190 | -0.0637 | 0.0125 | 0.0314 |
|  |  |  |  |  |  |
| **Antioxidants** |  |  |  |  |  |
| GSH + GSSG | 0.2663 | 0.4590 | 0.4261 | -0.1275 | 0.7161 |
| GSH | -0.5472 | 0.7460 | 0.2171 | -0.0963 | 0.2557 |
| GSSG | 0.7934 | -0.5428 | 0.0338 | 0.0253 | 0.1801 |
| GSH/(GSH + GSSG) | -0.7741 | 0.5824 | -0.0052 | -0.0333 | -0.1482 |
| ASC + DHA | 0.9753 | 0.1176 | -0.0949 | -0.0502 | -0.0101 |
| ASC | 0.9728 | 0.1234 | -0.0949 | -0.0511 | -0.0122 |
| DHA | 0.9897 | 0.0693 | -0.0946 | -0.0418 | 0.0072 |
| ASC/(ASC + DHA) | -0.8894 | 0.1344 | -0.0072 | -0.1005 | -0.1324 |
|  |  |  |  |  |  |
| **Eigen value** | 40.781 | 3.667 | 1.548 | 1.293 | 1.108 |
| **Variation percent (%)** | 81.561 | 7.334 | 3.095 | 2.587 | 2.216 |

**Additional file 4: Table S3.** PCA for physiological parameters of roots. -D, enzyme activity expressed on a dry weight (DW) basis; -P, enzyme activity expressed on a protein basis.

| **Variables** | **PC1** | **PC2** | **PC3** | **PC4** |
| --- | --- | --- | --- | --- |
| **Mg** | -0.9662 | -0.0566 | 0.1588 | 0.0616 |
|  |  |  |  |  |
| **Electrolyte leakage, ROS production rates, MDA and MG** | | |  |  |
| Electrolyte leakage | 0.9849 | -0.1243 | -0.0185 | -0.0169 |
| Superoxide anion production rate | 0.9834 | 0.0773 | 0.1346 | 0.0626 |
| H_2_O_2_ production rate | 0.9017 | -0.1120 | -0.0678 | 0.1423 |
| MDA | 0.9966 | -0.0497 | 0.0489 | -0.0309 |
| MG | 0.9478 | 0.2093 | 0.1734 | 0.0101 |
|  |  |  |  |  |
| **Antioxidant enzymes** |  |  |  |  |
| APX-D | 0.9785 | 0.0740 | 0.1858 | -0.0360 |
| APX-P | 0.9797 | 0.0714 | 0.1812 | -0.0319 |
| MDHAR-D | 0.9376 | 0.0526 | -0.2417 | 0.1800 |
| MDHAR-P | 0.9473 | 0.0530 | -0.2189 | 0.1697 |
| DHAR-D | 0.6853 | -0.1249 | 0.6551 | -0.1476 |
| DHAR-P | 0.7322 | -0.1143 | 0.6154 | -0.1463 |
| SOD-D | 0.9613 | -0.0250 | -0.0479 | 0.1554 |
| SOD-P | 0.9639 | -0.0241 | -0.0439 | 0.1519 |
| GuPX-D | 0.7330 | 0.6246 | -0.1811 | 0.1647 |
| GuPX-P | 0.7750 | 0.5838 | -0.1622 | 0.1563 |
| CAT-D | -0.9064 | 0.0441 | -0.0168 | 0.2943 |
| CAT-P | -0.8668 | 0.0624 | -0.0165 | 0.3424 |
|  |  |  |  |  |
| **S metabolism-related enzymes** |  |  |  |  |
| GST-D | 0.9936 | 0.0295 | 0.0858 | 0.0240 |
| GST-P | 0.9938 | 0.0282 | 0.0863 | 0.0253 |
| GR-D | 0.9424 | -0.2655 | -0.0092 | -0.0108 |
| GR-P | 0.9447 | -0.2613 | -0.0060 | -0.0080 |
| ATPS-D | 0.7520 | 0.0102 | -0.5875 | -0.0753 |
| ATPS-P | 0.8687 | 0.0122 | -0.4435 | -0.0330 |
| APR-D | -0.9671 | 0.1579 | -0.1105 | 0.0838 |
| APR-P | -0.9552 | 0.1916 | -0.1213 | 0.0970 |
| GlPX-D | 0.9796 | 0.0753 | -0.1534 | 0.0882 |
| GlPX-P | 0.9812 | 0.0726 | -0.1436 | 0.0895 |
| CS-D | -0.9172 | -0.2004 | 0.1307 | 0.2347 |
| CS-P | -0.8803 | -0.2396 | 0.1695 | 0.2732 |
| γGT-D | -0.9799 | -0.1360 | 0.0564 | 0.0770 |
| γGT-P | -0.9411 | -0.2031 | 0.1435 | 0.1423 |
| γGCS-D | 0.9853 | 0.0071 | 0.1000 | 0.0482 |
| γGCS-P | 0.9871 | 0.0091 | 0.0982 | 0.0446 |
| GS-D | -0.7931 | 0.3190 | 0.1937 | 0.4540 |
| GS-P | -0.6900 | 0.3958 | 0.2337 | 0.5230 |
| SiR-D | -0.9833 | -0.1278 | -0.0224 | -0.0289 |
| SiR-P | -0.9785 | -0.1474 | -0.0127 | -0.0286 |
|  |  |  |  |  |
| **Gly I and Gly II** |  |  |  |  |
| Gly I-D | -0.9572 | 0.1041 | -0.0357 | -0.1823 |
| Gly I-P | -0.9424 | 0.1323 | -0.0198 | -0.2082 |
| Gly II-D | 0.9710 | -0.0227 | 0.0886 | 0.0322 |
| Gly II-P | 0.9758 | -0.0204 | 0.0901 | 0.0305 |
|  |  |  |  |  |
| **Antioxidants** |  |  |  |  |
| GSH + GSSG | 0.3091 | 0.8896 | 0.1880 | -0.1085 |
| GSH | -0.4335 | 0.8654 | 0.1101 | -0.2095 |
| GSSG | 0.8197 | -0.5033 | -0.0077 | 0.2022 |
| GSH/(GSH + GSSG) | -0.7507 | 0.6242 | 0.0003 | -0.1925 |
| ASC + DHA | -0.9857 | -0.0713 | -0.1070 | -0.1020 |
| ASC | -0.9858 | -0.0751 | -0.1067 | -0.0977 |
| DHA | -0.9814 | -0.0252 | -0.1095 | -0.1528 |
| ASC/(ASC + DHA) | -0.8922 | -0.3522 | -0.1280 | -0.0272 |
|  |  |  |  |  |
| **Eigen value** | 41.0420 | 3.8815 | 2.0523 | 1.3766 |
| **Variation percent (%)** | 82.0840 | 7.7630 | 4.1046 | 2.7532 |
